# Autism Spectrum Disorder and auditory sensory alterations: a systematic review on the integrity of cognitive and neuronal functions related to auditory processing

**DOI:** 10.1007/s00702-023-02595-9

**Published:** 2023-03-14

**Authors:** Ana Margarida Gonçalves, Patricia Monteiro

**Affiliations:** 1 Life and Health Sciences Research Institute, School of Medicine, University of Minho, Campus de Gualtar, 4710-057 Braga, Portugal; 2 ICVS/3B’s—PT Government Associate Laboratory, 4710-057 Braga/Guimarães, Portugal; 3 Experimental Biology Unit, Department of Biomedicine, Faculty of Medicine, University of Porto, Porto, Portugal

**Keywords:** Autism spectrum disorder (ASD), Auditory, Sensory, Neuroscience, Patients

## Abstract

Autism Spectrum Disorder (ASD) is a neurodevelopmental condition with a wide spectrum of symptoms, mainly characterized by social, communication, and cognitive impairments. Latest diagnostic criteria according to DSM-5 (Diagnostic and Statistical Manual of Mental Disorders, Fifth Edition, 2013) now include sensory issues among the four restricted/repetitive behavior features defined as “hyper- or hypo-reactivity to sensory input or unusual interest in sensory aspects of environment”. Here, we review auditory sensory alterations in patients with ASD. Considering the updated diagnostic criteria for ASD, we examined research evidence (2015–2022) of the integrity of the cognitive function in auditory-related tasks, the integrity of the peripheral auditory system, and the integrity of the central nervous system in patients diagnosed with ASD. Taking into account the different approaches and experimental study designs, we reappraise the knowledge on auditory sensory alterations and reflect on how these might be linked with behavior symptomatology in ASD.

## Introduction

### Autism spectrum disorder

Autism spectrum disorder (ASD) is a neurodevelopment condition characterized by deficits in social communication and interaction, and restricted/repetitive behavioral features (Peça et al. 2011), showing first symptoms typically around three years old (Robertson and Baron-Cohen 2017). In 2018, the Centers for Disease Control and Prevention (CDC) reported approximately one in 44 children diagnosed with ASD, with a four times higher prevalence in boys versus girls. There is a wide variation in the type and severity of symptoms in ASD. Unlike other diagnoses, such as a specific phobia, it is not easy to draw a straight line from symptoms to diagnostic criteria. Each neurodivergent individual with ASD is unique, and the diagnosis is based on behavioral observation.

### ASD diagnosis evolution: DSM-5 and ICD-11

The year 2013 was an important landmark for Autism conceptualization, with the release of the latest version of the Diagnostic and Statistical Manual of Mental Disorders (DSM–5) (American Psychiatric Association (APA) 2013). Before DSM-5, there were poor diagnostic criteria with limited reliability in assigning subcategory diagnosis (Walker et al. 2004). The diagnosis of autism was categorized by subcategories (e.g., autistic disorder, Asperger’s disorder, and pervasive developmental disorder not otherwise specified). With the fifth edition of DSM, there was an important shift in the conceptualization of dimension: Autism became a single diagnosis based on multiple dimensions. The DSM-5 refers to Autism as a Spectrum Disorder, embracing an umbrella of symptoms with wide variety and severity levels. The new diagnosis of ASD includes persistent deficits in social communication and social interaction across multiple contexts, and restricted and repetitive patterns of behavior, interests, or activities (American Psychiatric Association (APA) 2013). These symptoms are present in early developmental period, significantly interfering with individuals’ daily functioning (American Psychiatric Association (APA) 2013).

In 2018, the World Health Organization updated the classification of autism in the International Classification of Diseases (ICD-11) (Organization 2018), to be more in line with DSM-5. The latest version of ICD-11 came into effect on January 2022 (Organization 2018). Both authoritative guidebooks used by medical professionals for the diagnosis and treatment of diseases and disorders collapse autism into a single diagnosis of Autism Spectrum Disorder, embracing the same two major aspects: difficulties in initiating and sustaining social communication and social interaction, and restricted interests and repetitive behaviors (Rosen et al. 2021).

The evolution of Autism criteria diagnosis reflects the evolving concern of clinicians and, particularly, researchers. If the diagnostic criteria are not easily well defined, people with autistic-like traits might be included, leading to misleading clinical cohorts that might hinder a clear understanding of autism neurobiology. As an example, as DSM-IV (American Psychiatric Association (APA) 1994), the previous version of the International Classification of Diseases, ICD-10 (Organization 1993), included a third category for language problems. The ICD-10 subdivided communication and social interaction into different clusters. Given that clinicians found it hard to categorize symptoms as either, both DSM-5 and ICD-11 now combine social and language deficits into a single measure. These deficits seem to be interrelated, being understood that a child with limited language or communication problems would have limited social interaction. However, the cause of these communication and language impairments is still unclear.

### Sensory processing in ASD

Over the years, the focus of ASD diagnosis was mainly related to cognitive, communication, and social impairments. But more recently, the criteria of diagnosis started to include another feature: the sensory processing domain (Robertson and Baron-Cohen 2017). The DSM-5, and more recently the ICD-11, now include sensory hyper- and hypo-sensitivities as part of the restricted and repetitive behavior domain (American Psychiatric Association (APA) 2013; Organization 2018). Atypical responses to sensory stimuli can also help to differentiate ASD from other developmental disorders (Stewart et al. 2016).

Sensory integration is a neurobiological process that refers to the integration and interpretation of sensory stimuli from surrounding context to the brain. Atypical sensory experience seems to occur in 85% of individuals with ASD and can be noticeable early in development (Robertson and Baron-Cohen 2017). Symptoms can include hypersensitivity, avoidance, diminished responses, or even sensory seeking behavior (Robertson and Baron-Cohen 2017; Sinclair et al. 2017). These alterations in sensory processing may interfere with the typical development of higher order functions such as social communication, which requires quick, accurate integration of sensory cues in real time (Robertson and Baron-Cohen 2017; Siemann et al. 2020). Many studies have looked into multisensory processing, highlighting the role of temporal binding windows as a critical factor in information integration (Robertson and Baron-Cohen 2017). The concept of temporal binding window refers to a window of time where specific modalities are perceptually bound (Hillock et al. 2011). A recent review from Siemann and colleagues describes numerous findings related to the presence of multisensory and temporal processing deficits in individuals with ASD (2020) (Siemann et al. 2020), suggesting atypical multisensory temporal processing with increased stimulus complexity. Although atypical sensory processing can be present across several sensory domains, atypical behavioral response to environmental sounds is among the most prevalent and disabling sensory feature of autism, with more than 50% of individuals exhibiting impaired sound tolerance (Williams et al. 2021).

### Auditory sensory processing in ASD

Many individuals diagnosed with ASD have auditory sensitivities, and it is common to observe children with ASD covering their ears, even in the absence of salient background noise. Individuals with ASD can present hyper- or hypo-sensitivity to a variety of sensory stimuli, which can cause a wide range of behavioral manifestations and maladaptations (Sinclair et al. 2017). Children underresponsive to sensory input appear to be unaware of auditory stimuli that are salient to others. Studies have shown that pronounced to profound bilateral hearing loss or deafness seems to be present in 3.5% of all cases, and hyperacusis (increased sensitivity or decreased tolerance to sound) affects nearly 18–40% of children with autism (Williams et al. 2021; Rosenhall et al. 1999; Wilson et al. 2017). Individuals with auditory hypersensitivity can notice auditory stimuli at intensity levels that are not salient or would not trouble others, which may cause sensory overload. For a person experiencing sensory overload, everyday sounds can be unpleasant and overwhelming, and that may lead to poor emotional and social regulation (Wilson et al. 2017), with impairments at the level of filtering out irrelevant input. The appropriate filtering of sensory information is crucial for healthy brain function, allowing salient awareness and focus on relevant social cues. Deficits in auditory processing can thus affect behavior, with profound effects on a person’s life, especially in the development of higher level skills such as social communication. However, the knowledge about phenomenology or neurocognitive underpinnings that underlie these auditory sensory processing deficits is still scarce. In fact, many studies have been performed before DSM-5 criteria, having a confound effect of potential misdiagnosis or comorbidity with other disorders.

To avoid such potential confounds, we opted for a systematic review of auditory sensory function in patients with ASD, considering studies performed between 2015 and 2022 (2 years after DSM-5 implementation), thus considering the recent adaption of autism diagnosis as a spectrum disorder.

The following questions will be addressed:

A. What is known about the integrity of Cognitive Function in auditory-related tasks?
B. What is known about the integrity of Peripheral Auditory System in auditory-related tasks?
C. What is known about the integrity of Central Auditory Nervous System in auditory-related tasks?

## Methods

### Protocol

This systematic review follows the Preferred Reporting Items for Systematic Reviews and Meta-Analyses (PRISMA) guidelines (Page et al. 2021), and review methods were established before initiating literature screening.

### Search strategy and screening criteria

The search strategy started in September 2021 and used MeSH terms from PubMed. Used databases: PubMed, Web of Science (all databases/collections), Scopus, and Scielo. Search terms included “autis*” AND “*auditory*”, and were limited to studies published between 2015 and March of 2022. The minimum year of 2015 was considered as the mark of first experimental studies using DSM-5 as a diagnosis of Autism Spectrum Disorders (American Psychiatric Association (APA) 2013).

### Study design

Studies were eligible if they consisted of original and reported data, with comparison group design, written in English and published in peer-reviewed journals. Conference abstracts, books and documents, editorials, letters, pilot studies, case studies, reviews, and meta-analyses were excluded.

### Participants

Only studies with human participants with a clear diagnosis of ASD were considered. To avoid confounding results, studies with ASD participants that were also diagnosed with other symptomatology and/or disorders were excluded: epilepsy, Williams Syndrome, ADHD, Rett Syndrome, Angelman Syndrome, Fragile X Syndrome, etc. Participants diagnosed with ASD in early childhood but without current ASD symptoms, or participants with the diagnosis based on the DSM-IV without other assessments of diagnosis validation (e.g., ICD-11 and ADOS) were also excluded. No restrictions of age, gender, racial, ethnic, and socioeconomic groups were made.

### Outcomes

Studies that did not include relevant outcome data were excluded (e.g., ASD parents’ symptomatology; studies focused on visual processing that used auditory cues in the task that are not referred to in the results; studies focused on pilot training or intervention programs).

## Results and critical discussion

Most of the tools used to confirm ASD diagnosis in the studies included on this review were: Short-Sensory Profile (SPP) (Dunn 1999), Childhood Autism Rating Scale (CARS) (Schopler et al. 2010), Autism Diagnostic Observation Schedule, Second Edition (ADOS-2) (Lord et al. 2012), and the Peabody test (Dunn and Dunn 1965). To estimate global intellectual ability (full-scale IQ), most tests used were the Wechsler Adult Intelligence Scale or the Wechsler Intelligence Scale for Children (Wechsler and Kodama 1949), and Raven’s Colored Progressive Matrices Test (Measso et al. 1993). Even if there is no official diagnosis for ASD being described as low or high functioning, some papers identified some ASD groups as being high-functioning autism (usually subjects with an IQ mean higher than 85).

### Integrity of cognitive function in auditory-related tasks

Cognitive function can be assessed by mental processes such as perception, attention, memory, decision-making, or language comprehension, and in the domain of social cognition (e.g., theory of mind, social and emotional processing). People with ASD have a profile of cognitive strengths and weaknesses, demonstrated by different levels of deficits in social and non-social cognition patterns. To understand how auditory input might influence cognitive function in ASD, it is useful to assess cognitive integrity at several domains, while performing auditory-related tasks.

#### Attention, detection, and discrimination

Communication in everyday life depends crucially on the ability to detect stimuli and dynamically shift attention between competing auditory streams. We use attention to direct our perceptual systems toward certain stimuli for further processing. Difficulties in attention processes can directly affect social interaction and communication abilities. When compared to neurotypical controls, individuals with ASD seem to have poorer performance in many auditory attention tasks: divided attention (ability to attend to two or more stimuli at the same time), sustained attention (ability to attend stimulus over longer periods), selective attention (ability to ignore details of stimulus not attended), and spatial attention (selecting a stimulus based on its spatial location). Main findings are summarized in Table 1.

**Table 1:** Summary results of attention studies, organized by type of attention

Type of attention	Study	IQ assessment		Mean age (SD)	Experiment	
		Test	Cognitive standard scores Mean (SD)	ASD	Task	Results ASD
DIVIDED	Foster et al. (2016)	WASI	IQ > 70	12.9 y (2.5)	Direct and Divided attention	Less sensitive to global interference
SUSTAINED	Crasta et al. (2020)	WASI	112 (12.37)	8.24 (1.39)	TEA-Ch	TEA-Ch: worst scores in Sustained and Control attention
					Sustained and control attention	
	Pastor-Cerezuela et al. (2020)	Raven, Peabody	Non-verbal: 100.88 (lv 2)	81.20 m (11.18)	Nepsy-II	Sustained-attention: worst performance
			Verbal: 72.68 (18.19)		Maintain *vs* sustained attention	
SELECTIVE	Emmons et al. (2022)	WASI-II	102.36 (14.33)	21.75 y (0.64)	Selective *vs* maintain-listening	No differences between hold and maintain tasks worst accuracy at both compared with TD
SPATIAL	Soskey et al. (2017)	WISC-IV	111 (12.30)	13.78 y (1.93)	Target and distractor sounds	Diffuse auditory spatial attention across all stimulus types more diffuse: front target | increased responding to sounds at adjacent non-target locations

*DIVIDED attention* ability to attend two or more stimuli at the same time, *SUSTAINED attention* ability to attend stimulus over long periods, *SELECTIVE attention* ability to ignore details of stimulus not attended, *SPATIAL attention* involves selecting a stimulus on the basis of its spatial location

*Raven* Raven Colored Progressive Matrices Test, *Peabody *Peabody picture vocabulary test, *TEA-Ch *Test of Everyday Attention for Children, *lv 2* level 2 of severity, *vs* versus, *y* years, *m* months

Low-level auditory discrimination ability seems to vary widely within ASD. When different paradigms were compared, no differences were found for adults with ASD regarding selective attention (maintain vs switch tasks) (Emmons et al. 2022). However, infants and children with ASD presented poorer performances for sustained and control attention, attentional disengagement, and reorienting (Keehn et al. 2021), when compared with typically developing peers (TD). Spatial attention, a key component for social sound orientation, was measured by looking at central or peripheral locations of target sounds location while ignoring nearby sounds (Soskey et al. 2017). Adults diagnosed with ASD show diffuse auditory spatial attention independently of stimulus complexity: simple tones, speech sounds (vowels), and complex non-speech sounds (Soskey et al. 2017). Interestingly, children with ASD show more diffuse auditory spatial attention, indicated by increased responding to sound at adjacent non-target locations (Soskey et al. 2017). Subjects with ASD also seem to be less sensitive to global interference, or mixed cues, when compared with just one cue (local targets processed slower due to the presence of inconsistent global information) (Foster et al. 2016).

#### Responses to sensory stimuli: visual versus auditory—distinctive multisensory integration

Information from different sensory modalities, such as auditory and visual stimuli, can be easily integrated simultaneously by the nervous system. The visual sensory system of ASD individuals seems to be similar to TD peers, as opposed to their auditory system (Little et al. 2018). Different sensory modalities combined can create multisensory facilitation. As an example, speech perception is boosted when a listener can see the mouth of a speaker and integrate auditory and visual speech information. However, multisensory integration (MSI) is thought to be distinctive in ASD (Ainsworth et al. 2020) (Table 2), with temporal processing deficits that are not generalized across multiple sensory domains (Ganesan et al. 2016).

**Table 2:** Summary results of multisensory integration, organized by level of perceptual process

Perceptual process	Experiment				IQ assessment		Mean age (SD)
	Study	Paradigm	Stimuli	Results	Test	Cognitive standard scores Mean (SD)	ASD
LL	Poole et al. (2021)	Determine whether the stimulus was continuous or pulsed on each trial	Visual, tactile and auditory	ASD: shift costs were observed for each target modality in participant response times—> conditions called target – previous target ASD: largest for auditory targets (auditory repeat trials) when compared with visual and tactile targets	Weschler	118.41 (10.15)	30.58 (7.40)
LL	Ainsworth et al. (2021)	Target detection task	Auditory (A; 3500 Hz tone), visual (V; white disk ‘flash’) or audiovisual (mix of both)	ASD showed reduced multisensory facilitation compared to NT participants in a simple target detection task, void of social context	WISC-IV	112.85 (17.89)	11.91 (2.00)
					WASI-II	107.16 (11.57)	19.05 (4.10)
LL	Ostrolenk et al. (2019)	RTs on the audiovisual condition	Visual and auditory non-social stimuli (i.e., flashes and beeps)	MSI of simple information, void of social content or complexity, altered in autism	Weschler	102.95 (13.71)	19.21 (4.71)
LL	Stewart et al. (2016)	Determine whether the stimulus was high (tone/visual position) or low	Unisensory auditory, unisensory visual, and bisensory (congruent auditory and visual stimuli	Reduced response times for bisensory compared to unisensory trials were seen in both ASD and control groups	WASI	109.4 (15.3)	12.8 (2.9)
LL + HL	Righi et al. (2018)	Eye tracking (sensitivity to temporal asynchronies in a speech processing)	Videos synchronized (or not) with audio	ASD: failed to demonstrate sensitivity to asynchronies of 0.3 s, 0.6 s, or 1.0 s	Bayley	69.73 (25.43)	5.12 (1.53)
				Correlated with language abilities—measured by PLS	WPPSI		
					Stanford Binet		
LL + HL	Noel et al. (2017)	Simultaneity judgment (asynchronous audiovisual stimuli of varying levels of complexity)	Simple and non-linguistic stimuli (i.e., flashes and beeps, hand-held tools) and speech stimuli	ASD fail to rapidly recalibrate to audiovisual asynchronies simple and non-linguistic stimuli, but exhibit comparable rapid recalibration for speech stimuli	WASI-2	110.23 (14.05)	
HL	Foss-Feig et al. (2017)	Psychophysical gap detection task	Visual and auditory	Domain-specific impairment in rapid auditory temporal processing in ASD that is associated with greater difficulties in language processing	Weschler	115.96 (17.4)	11.94 (1.3)
					Auditory task		
HL	Smith et al. (2017)	Temporal window of Integration	Basic speech (consonant–vowel utterances) and object stimuli (bouncing ball)	ASD: less tolerance of asynchrony for speech stimuli compared to object stimuli	Weschler	111.45 (15.57)	14.55 (2.18)
HL	Chahboun et al. (2016)	Cross-modal sentence- picture matching task	Figurative expressions and their target figurative meaning represented in images	ASD displayed higher error rates and greater reaction latencies in the auditory modality compared to the visual stimulus presentation modality	WISC-IV	110.71 (14.58)	11.3 (0.96)
					WAIS-IV	108.3 (13.39)	18.1 (1.65)
HL	Turi et al. (2016)	Instantaneous adaptation to audiovisual asynchrony	Visual and auditory stimuli, varying in asynchrony over a wide range, from 512 ms auditory-lead to 512 ms auditory-lag	Typical adults showed strong adaptation effects	Weschler	112.0 (10.32)	29.2 (5.2)

*LL* Low Level Perceptual Process, *HL* High Level Perceptual Process, *Bayley* Bayley Scales of Infant Development, *Stanford Binet* Stanford Binet Intelligence Scales, *Weschler* Weschler Full-Scale IQ, *WPPSI* Wechsler Preschool and Primary Scale of Intelligence-III, *PLS* Preschool Language Scales

##### Low-level perceptual processes

An experimental design based on Miller’s race model (Miller 1982) was tested by assessing the accuracy and reaction times of unisensory and bisensory (visual and auditory) trials and by comparing children with ASD with typically developing peers (TD) as a control group (Stewart et al. 2016). Findings did not support impaired bisensory processing for simple non-verbal stimuli in high-functioning children with ASD (Stewart et al. 2016). Reduced bisensory facilitation was found for both ASD and TD groups, suggesting intact low-level audiovisual integration. Another study assessed the reaction time of target stimuli detection task (auditory, 3500 Hz tone; visual, white disk ‘flash’; and audiovisual, simultaneous tone and flash) by comparing younger (age 14 or younger) and older participants (age 15 and older) (Ainsworth et al. 2020). The authors found greater multisensory reaction time facilitation for neurotypical (NT) adults, increased for older participants, and reduced multisensory facilitation for ASD, both in younger and older participants.

##### High-level perceptual processes

Audiovisual integration of basic speech and object stimuli was compared in children and adolescents diagnosed with ASD (Smith et al. 2017). To measure audiovisual perception, a temporal window of integration was established, where individuals identify which video (temporally aligned or not) matched the auditory stimuli. Results showed similar tolerance of asynchrony for the simple speech and object stimuli for controls, while ASD adolescents showed decreased tolerance of asynchrony for speech stimuli. These results were associated with higher levels of symptom severity (Smith et al. 2017). A similar result of instantaneous adaptation to audiovisual asynchrony was found in ASD adults (Turi et al. 2016). This means that reduced adaptation effect in ASD individuals, and poorer multisensory temporal acuity, may be hindering speech comprehension and consequently affecting social communication.

Social interaction implies attentional shift, such as joint attention (when a person intentionally focuses attention on another person). A study tested whether impairments in joint attention were influenced by self-relevant processing. Results showed that gaze-triggered attention is influenced by self-relevant processing and symptom severity in ASD individuals, with reduced cueing effect to voice compared to tone targets (Zhao et al. 2019).

The stimulus presentation modality can lead to differences in figurative language comprehension, even with high-verbal ASD individuals (matched with TD peers on intelligence and language level) (Chahboun et al. 2016). When visual and auditory modalities are compared, individuals with ASD present the poorest performances in the auditory modality, showing difficulties to understand culturally based expressions (Chahboun et al. 2016). Greater difficulties in language processing seem to be correlated with higher auditory gap detection thresholds (minimum interval between sequential stimuli needed for individuals to perceive an interruption between the stimuli) (Foss-Feig et al. 2017).

#### The influence of noise on cognitive performance

Multisensory facilitation can be highly valuable, especially in noisy environments. A diminished capacity to integrate sensory information across modalities can potentially contribute to impaired social communication.

Cognitive performance in ASD was assessed with experimental manipulation of noise (quiet and 75 dB gated broadband noise) adding different levels of task difficulty (easier and harder) (Keith et al. 2019). Results show that for NT adolescents, it is easier to adapt to the effect of noise. In contrast, the ASD group shows a detrimental effect of noise and increased arousal in the harder condition (Keith et al. 2019). Children with ASD who attend more time to the stimulus, such as looking longer to the speaker’s face, show better listening performance (Newman et al. 2021). When different levels of signal-to-noise ratio (SNR) are compared, ASD and NT groups exhibited greater benefit at low SNRs relative to high SNRs in phoneme recognition tasks (Stevenson et al. 2017). The ASD group shows a reduced performance in both auditory and audiovisual modalities for whole-word recognition tasks with high SNRs (Stevenson et al. 2017), when compared with TD peers. High-functioning ASD adults show a speech comprehension rate of nearly 100% in the absence of noise, similar to IQ-matched controls (Piatti et al. 2021; Dwyer et al. 2021). However, under noise conditions, the ASD group presents worse speech comprehension and neural over-responsiveness (Piatti et al. 2021; Dwyer et al. 2021). Such results suggest that noise may increase the threshold needed to extract meaningful information from sensory inputs, affecting speech comprehension.

#### Increased auditory perceptual ability: differences in auditory stimuli detection and discrimination

Regarding auditory perceptual capacity, there is a wide variety of experimental designs, paradigms, and tested parameters, holding different results (Table 3). When ASD and TD individuals were compared, significant differences were found in the ASD group regarding several acoustic parameters, such as deficits in the intensity or loudness of stimuli, higher auditory duration discrimination threshold of stimuli, and enhanced memory for vocal melodies (Kargas and Lo 2015; Isaksson et al. 2018; Weiss et al. 2021). Musical and vocal timbre perception seems to be intact for older ASD participants (Schelinski et al. 2016), with one study showing decreased music ability, related with deficits at the level of working memory and hyperactivity/inattention of young ASD children (Sota et al. 2018).

**Table 3:** Summary results of auditory detection and discrimination studies, organized by year

Experiment				IQ assessment		Mean age (SD)
Study	Paradigm	Stimuli	Results	Test	Cognitive standard scores Mean (SD)	Or Mean age (range) ASD
Weiss et al. (2021)	Memory for vocal and instrumental melodies	Vocal (e.g.. *la, la*) and instrumental (piano, marimba) melodies	ASD: Enhanced Memory for Vocal Melodies	WASI	104.4 (19.2)	11.1 (1.4)
Schelinski et al. (2019)	Vocal Emotion Recognition Test, Vocal Pitch and Vocal Timbre Discrimination Test, Non‐vocal Pitch Perception Test	Auditorily presented words (65 dB)	Vocal emotion: impaired ASD	WAIS-III	110.31 (13.79)	33.75 (10.12)
			ND for ASD: pitch, timbre			
Germain et al. (2018)	Low-level pitch direction	Pairs of tones that differed in pitch and were presented at various temporal rates (pure sine tones + harmonics)	Pitch direction perception predicts global–local pitch processing (individual differences in low-level pitch direction ability predicted performance on the higher level global–local task, with a stronger relationship in ASD)	WASI	110.8	13.7 (2.3)
	High-level global–local task	Three-tone triplet sequences combined to form sequences of nine harmonic tones				
Isaksson et al. (2018)	Motor and perceptual timing	Free tapping, simultaneity judgment, auditory duration discrimination, and verbal duration estimation	Auditory duration discrimination thresholds: higher for ASD	WISC-V	102 (18)	11:0 (2:4)
		Dinosaur computer task	ASD with abnormalities in temporal processing tasks: motor timing, perceptual timing, and temporal perspective			Years:months
Sota et al. (2018)	Musical ability	MBEA	ASD: Musical ability decreased	Weschler	88.85 (12.24)	8.62 (2.47)
			Working memory and hyperactivity/inattention predicted musical ability			
Chowdhury et al. (2017)	Relationship between auditory pitch perception and verbal and non-verbal cognitive abilities in ASD versus TD children	Pairs of tones that differed in pitch and were prompted to choose whether the second tone had a lower or higher pitch	No group differences in performance	Weschler	110.8 (18.3)	
			Significant variability in performance on the auditory tasks			
			Auditory perception is related to non-verbal reasoning rather than verbal abilities			
Park et al. (2017)	Visual orientation discrimination in the presence of varying levels of external noise	Coarse orientation discrimination task sandwiched by noise stimuli	ASD: increased internal noise and worse external noise filtering	Weschler	110.14	13.49 (2.08)
		Measuring perceptual sensitivity across varying levels of external noise	Internal noise significantly correlating with ASD symptoms (ADOS)			
Remington and Fairnie (2017)	Detection and identification tasks	Exp1. dual-task paradigm (target sounds, dog bark; + non- target, duck)	ASD better at detecting additional unexpected and expected sounds	Weschler	110 (13)	30 (3.6)
	(Highlight both the benefits and disadvantages of increased capacity)	Exp2. 69 s auditory scene	Increased distraction and superior performance			
Haigh et al. (2016)	Auditory modulation-depth discrimination task	Visual (grating patches) + Auditory (tones)	No evidence of atypical sensory function or atypical attentional modulation	Weschler	114.8 (13.4)	27 (21–42)
Schelinski et al. (2016)	Unfamiliar voice discrimination test, Famous voice recognition test, Acoustic voice feature processing test	UVL (voice-face learning; voice-name learning; voice-color learning); ACF (pitch and timbre)	ASD: difficulties with discriminating, learning, and recognizing unfamiliar voices (particularly pronounced for learning novel voices)	WAIS	107.38 (17.55)	33.75 (10.12)
			ASD: deficit in vocal pitch perception			
			ASD: intact acoustic processing (musical pitch, musical, and vocal timbre perception)			
			Familiar voices: ND			
Mayer et al. (2016)	Different pitch discrimination trajectories	Complex tones and speech pitch	Pitch discrimination increased with age (TD). ASD: enhanced childhood and stable in adults	Ravens	34.67 (5–75th percentile)	126.07 m (47.53)
						165.64 (23.46)
		Monosyllabic words + pitch contours derived from these words				482.79 (136.00)
Boets et al. (2015)	Right versus left auditory cortex processing	Frequency discrimination and slow amplitude modulation (AM) detection versus gap-in-noise detection and faster AM detection	ASD: impaired frequency discrimination	WISC-III-NL	> 80	(12–19)
		Target right auditory cortex processing (frequency discrimination, 4 Hz AM) and left auditory cortex processing (gap-in-noise detection, 20 Hz AM)	Gap- in-noise detection thresholds: poorer temporal resolution for ASD			
			Not support the hypothesis of superior right and inferior left hemispheric auditory processing in ASD			
Jiang et al. (2015)	Discrimination and identification	Melodic contour and speech intonation	ASD: superior melodic contour identification but comparable contour discrimination	Ravens	119.12 (13.77)	9.41 (3.03)
			ASD performed worse than controls on both discrimination and identification of speech intonation	VIQ	116.06 (27.17)	
Kargas et al. (2015)	Loudness, pitch, duration (intensity, frequency and duration)	Standard pure tone (74 dB) and a probe tone (55 to 73.5 dB)	Significant deficits in ASD on all acoustic parameters	WASI	109.8 (18.2)	30.3 (10.4)
			Low-level auditory discrimination ability varies widely within ASD—> this variability relates to IQ level, and influences the severity of RRBs			

*Ravens* Ravens Progressive Matrices, *Weschler* Weschler Full-Scale IQ, *UFV* Unfamiliar voice discrimination test, *AVF* Acoustic voice feature processing test, *TD* typical development, *ND* no differences, *RRBs* restricted and repetitive behaviors, *MBEA* Montreal Battery of Evaluation of Amusia

One of the common parameters used to test auditory perceptual capacity is pitch detection, the degree of highness, or lowness of a tone. Studies have shown a heightened pitch detection in the ASD group, for expected and unexpected sounds (Remington and Fairnie 2017). Regarding the developmental trajectory of pitch perception, findings reveal enhanced pitch discrimination in childhood with stability across development in ASD (Mayer et al. 2014). Interestingly, this low-level auditory ability seems to vary widely within ASD, being related to IQ level and severity of symptoms (Kargas and Lo 2015). Adults diagnosed with ASD show superior pitch perception associated with sensory deficits (Mayer et al. 2014). Individual differences in low-level pitch discrimination tasks can predict performance on the higher level global–local tasks (Germain et al. 2018). Results seem to vary between detection and discrimination tasks across studies. A discrimination task assesses the ability to detect the presence of a difference between two or more stimuli. Interestingly, no differences were found between low and higher level pitch processing (Chowdhury et al. 2017) when ASD children were matched in terms of IQ levels and verbal and non-verbal cognitive abilities, as measured by WASI subtests yield (Wechsler and Kodama 1949). For ASD adults without intellectual impairment, when participants were asked to identify modulation-depth differences (e.g., − 3 dB), no differences were found in auditory modulation–depth discrimination tasks (Haigh et al. 2016). ASD adults showed difficulties in discriminating, learning, and recognizing unfamiliar voices (particularly pronounced for learning novel voices) (Schelinski et al. 2016). These results may allow us to speculate that an increased auditory capacity, such as heightened pitch detection, may lead to better performance at detection or discrimination tasks, but may also entail a sensory overload, greatly enhancing the difficulty of the task for different stimuli parameters.

#### Auditory processing and language-related impairments

Atypical sound perception and auditory processing deficits may underlie language and learning difficulties in ASD, impairing social communication skills. Studies have found that ASD patients tend to display worst performance in whispered speech compared to normal speech (Venker et al. 2019; Georgiou 2020) and several differences have been reported in terms of language processing and speech perception (Table 4).

**Table 4:** Summary results of language assessment, organized by year

Source	Experiment			Cognitive standard scores		Mean age ± SD
	Paradigm	Stimuli	Results ASD	Test	Mean (SD)	ASD
Georgiou (2020)	Identification of native vowel in normal and whispered speech	Greek vowels (/i e a o u/) embedded in a monosyllabic / pVs/ context	Slower in the whispered speech	Raven	> 40 out of 60	31.2 y (4.5)
			Children with weaker receptive language showed a smaller head start than children with stronger receptive language skills			
Venker et al. (2019)	Incremental language processing and receptive language (longitudinal)	Semantically-constraining verbs (e.g., Read the book) compared to neutral verbs (e.g., Find the book)	Head start when presented with semantically-constraining verbs	Mullen	77.06 (26.80)	56.15 m (3.94)
Noel et al. (2018)	Simultaneity judgment task to index their audiovisual temporal acuity for speech stimuli	Syllable /ba/ or /ga/ (audio and visual components)	Wider window of audiovisual temporal integration	TONI-4	106.34 (18.34)	12.20 y (3.75)
Stevenson et al. (2018)	Temporal order judgment task	White visual rings on a black background paired with auditory pure-tone beeps	Temporal processing abilities in children with autism contributed to impairments in speech perception	WASI-II	–	12.3 y (3.1)
Foss-Feig et al. (2017)	Visual and auditory temporal processing abilities	Gap detection tasks to measure gap detection thresholds	Higher auditory gap detection thresholds	Wechsler	115.96 (17.4)	11.94 (1.3)
			Correlated significantly with several measures of language processing			

*m* months, *y* years

The integration of information for successful language processing and speech comprehension relies on precise and accurate temporal integration of auditory and visual cues. Children with ASD seem to possess a wider window of audiovisual temporal integration (Stevenson et al. 2017; Noel et al. 2017), and higher auditory gap detection thresholds (Foss-Feig et al. 2017). Deficits at the level of temporal integration may contribute to impaired speech perception. Studies of auditory processing and language paradigms show that language processing can be facilitated in children with ASD using contextual references such as semantically-constrained verbs compared to neutral verbs (Venker et al. 2019).

Language components, such as phonemes, syntax, and context, work together with features to create meaningful communication among individuals. All languages use pitch and contour to carry information about emotions and to communicate non-verbally. The way we say something affects its meaning, just by using different emphasis and intonation. Phonological use of pitch dissimilarities is distinct between tone and non-tone languages at several levels of their phonological hierarchy (prosodic word, phonological phrase, intonational phrase, and utterance tiers) (Beckman and Pierrehumbert 1986). Tones are associated with lexical meaning to distinguish words. English, as a non-tone language, uses contrastive pitch specifications at a segmental level, to express syntactic, discourse, grammatical, and attitudinal functions. A tone language, like Mandarin, uses constructive pitch specifications at every level of the phonological hierarchy (Best 2019). Pitch processing was assessed in individuals who speak Mandarin, by testing melodic contour and speech intonation (Jiang et al. 2015). ASD individuals presented superior melodic contour but comparable contour discrimination, and when compared with controls, ASD performed worse on both identification and discrimination of speech intonation (Jiang et al. 2015), suggesting a differential pitch processing in music that does not compensate for speech intonation perception deficits.

#### Auditory processing and cognitive function assessment: EEG and ERP

Many studies focusing on language processing use electroencephalography (EEG) to measure brain activity in real time. EEG signals reflect electrical activity produced by the brain (brain waves). An approach known as event-related potential (ERP), uses EEG activity that is time-locked with ongoing sensory, motor, or cognitive events, to help identify and classify perceptual, memory, and linguistic operations (Sur and Sinha 2009). To avoid confounding effects in the interpretation of ERP results, most studies only include children with normal or corrected-to-normal vision, normal hearing, and absence of genetic or neurological disorders, history of seizures, or past head injury. To check for hearing disabilities, many studies also perform a hearing screening with pure-tone audiometry at 20 dB. However, there is a wide variation regarding the inclusion criteria of participants (Table 5).

**Table 5:** Summary results of ERP study designs, organized by year

Source	Field	*n*		Mean age (SD)		Diagnose confirmation tools	IQ assessment				Inclusion criteria		Medication
		ASD	TD	ASD	TD		Test	Cognitive standard scores			No history of		
								ASD: Mean (SD)	TD: Mean (SD)	Differences	Hearing Loss	Visual	Yes, if criteria
Borgolte et al. (2021)	Audiovisual speech perception	14	15	40.3 (8.9)	42.4 (12.7)	Autism spectrum empathy quotient	MWT-B	108 (8)	106.4 (5.5)	ND	–	Yes	–
Chen et al. (2021)	Phonetic encoding	24	24	7.60 (2.32)	7.46 (1.91)	ADOS-2	Wechsler VIQ	91.54 (14.65)	100.13 (10.99)	*	Yes	–	Yes
						GARS-2	Wechsler NVIQ	99.33 (13.40)	103.83 (10.50)	ND			
Dwyer et al. (2021)	Heterogeneity: individual differences	243	96	38.50 months (6.02)	37.09 months (6.46)	ADOS-G	MSEL DQ	65.25 (20.91)	106.37 (11.58)	***	–	–	–
						ADI-R							
Piatti et al. (2021)	Attentional orienting to sounds in speech	ASD/DD: 22	17	ASD/DD: 34.37 (7.11)	38.21 (10.99) months	ADOS-2	DQ score DD:	< 71	> 71	***	–	–	Included 1 participant
		ASD/noDD: 12		ASD/noDD: 39.62 (7.54)		M-SEL	DQ score noDD:	> 71					
Jamal et al. (2021)	Sensory habituation	13	22	Range 7.4–12.8	Range 7.1–12.8	ADOS-2	WISC-V NVIQ	104.42 (12.59)	116.90 (9.73)	ND	–	–	–
						SCQ							
Kadlaskar et al. (2021)	Tactile and auditory reactivity patterns	14	14	10.13 (1.9)	9.95 (1.36)	ADOS-2	Wechsler	98 (21) verbal	117 (11) verbal	**	Yes	**-**	*-*
						SP2	DAS-II	108 (18) non-verbal	117 (16) non-verbal	ND			
						SRS							
Dwyer et al. (2020)	Heterogeneity: individual differences	132	81			ADOS-G	MSEL DQ	64.83 (20.49)	106.36 (11.57)		–	–	–
						ADI-R							
Green et al. (2020)	Speech sound differentiation	15	10	ASD–LI: 7.38 (1.19)	8.50 (1.72)	CARS-2	CELF-5	ASD–LI: 98 (7.58)	111.40 (11.61)	*	Yes	–	–
				ASD + LI: 7.29 (1.70)				ASD + LI: 61.57 (15.74)		***			
Knight et al. (2020)	Predictive coding	21	19	14	15	ADOS- 2	Wechsler	100	117	*	Yes	Yes	Psychotropic medication included
Schwartz et al. (2020)	Own name	ASD-V: 27	27	ASD-V: 17.21 (2.08)	17.81 (3.00)	ADOS	Leiter	ASD-V: 109.63 (20.83)	–	–	Yes	–	–
		ASD-MLV: 20		ASD-MLV:16.81 (2.64)				ASD-MLV: 54.75 (20.24)					
van Laarhoven et al. (2020)	Predictive coding	29	29	18.64 (2.11)	18.93 (1.22)	ADOS	WAIS- IV-NL	103.03 (16.76)	112.07 (11.68)	ND	Yes	Yes	Yes
DiStefano et al. (2019)	Semantics	40	18	88.67 (22.04): VASD	91.61 (24.50)	SCQ	DAS-II Verbal IQ	118.28 (16.19)	75.97 (24.71) | 27.17 (14.32)	***	Yes	Yes	–
				92.42 (22.53): MVASD		ADOS	DAS-II Non-Verbal IQ	115.11 (16.50)	86.52 (32.81) | 42.89 (19.69)	***			
Grisoni et al. (2019)	Semantic understanding and predictive coding	20	22	38 (10.3)	31.9 (11.1)	Autism Spectrum Quotient questionnaire	LPS-3 Test	119.5 (8.4)	116.8 (9.5)	ND	Yes	Yes	-
Patel et al. (2019)	Prosody	19 (12 M)	20 (12 M)	17.22 (6.30)	14.99 (7.60)	ADOS-2	Wechsler	98.11 (22.55)	116.92 (13.44)	**	Yes	–	–
						ADI-R							
Ruiz‐Martínez et al. (2019)	Habituation and auditory discriminative process	16	15	8.96 (1.01)	8.86 (1.77)	ADOS-G	KBIT	50.26 (38.99)	85 (15.99)	***	Yes	–	–
Thomas et al. (2019)	Own name	19	13	52.29 (9.43)	51.22 (9.05)	ADOS-2	MSEL verbal t-score	36.08 (10.79)	53.84 (7.34)	***	Yes	Yes	–
						SCQ	MSEL non-verbal t-score	36.04 (12.73)	55.53 (8.00)	***			
Zhang et al. (2019)	Non-Speech and Speech Pitch Perception	16	16	10.42 (2.12)	9.48 (0.86)	~	Raven	20.13 (2.50)	21.13 (1.75)	ND	–	–	–
Arnett et al. (2018)	Implicit language learning and receptive language ability	27	76	12.12 (2.9)	13 (2.3)	ADI-R	DAS-II	88.45 (28.55)	113.59 (15.99)	**	–	–	–
						ADOS-2							
Charpentier et al. (2018)	Prosody	15	15	10.0 (1.4)	9.8 (1.4)	ADI-R	Wechsler	75 (29)	118 (19)	*	Yes	–	Included 4 participants
		16	16	26.2 (6.8)	26.2 (6.4)	ADOS-2		95 (18)	116 (16)	*			
Foss-Feig et al. (2018)	Temporal processing: silence gaps	15	17	11.86 (1.4)	12.23(1.2)	ADI-R	Wechsler	118.27 (13.8)	112.56 (12.6)	ND	Yes	Yes	Yes
						ADOS-2	Intact cognitive skills (IQ > 70)						
Goris et al. (2018)	Sensory Prediction Error	18	24	Adults	Adults	ADOS	WAIS-III	> 80	> 80	ND			
Huang et al. (2018)	Sensitivity to duration contrasts in speech and non-speech contexts	22	20	9.6 (1.88)	9.4 (1.71)	GARS-2	Raven	74 (23) tone	103 (17) tone	***	–	–	–
						ADOS		78 (15) vowel	101 (16) vowel	***			
Hudac et al. (2018)	Cognitive response to environmental change	102	31	12.29 (3.56)	13.27 (2.34)	ADI-R	Wechsler VIQ	81.3 (30.58)	115.06 (13.91)	***	–	–	–
						ADOS-2	Wechsler NVIQ	82.26 (30.08)	115.71 (16.39)	***			
Lindström et al. (2018)	Prosody	15	16	10.4	10.1	ICD-10	WISC-III	98 (12.89)	108 (12.9)	ND	Yes	–	Yes
Lodhia et al. (2018)	Auditory spatial cues	15	15	25.80 (6.81)	27.07 (5.80)	DSM-V	Wechsler	122.93 (12.83)	129.07 (7.40)	ND	Yes	–	Yes
Zhang et al. (2018)	Lexical stress	15	16	10.04 (1.53)	9.48 (0.86)	EYAB	Non-verbal IQ	–	–	ND	–	–	–
Bidet-Caulet et al. (2017)	Voice perception	16	16	10 years 6 months(1 year 5 months)	10 years 5 months (1 year 5 months	ADOS-G	EDEI-R	69 (25) verbal		*	Yes	–	–
						ADI-R	WISC	85 (18) non-verbal		*			
Galilee et al. (2017)	Detection and discrimination of speech and non-speech sounds	14	14	61 months (8.8)	50 months (11)	ADOS-G	BAS-II verbal	> 70	> 70	ND	Yes	Yes	–
						SCQ							
Wang et al. (2017)	Speech-specific categorical perception	16	15	10.4	10.3	GARS-2	Raven	83.7 (11.7)	86.3 (6.61)	ND	Yes	–	–
Karhson and Golob (2016)	Top–down and bottom–up attentional processes	12	13	22.5 (4.1)	22.8 (5.1)	ADOS	KBIT-2	105.08 (19.25)	101.25 (10.37)	ND	Yes	–	Yes
						ADI-R	Raven						
Key et al. (2016)	Speech sound differentiation	24	18	6. 71 (1.34)	7.14 (1.45)	ADI-R	K-BIT2	93.88 (17.41)	110.44 (13.05)	***	Yes	Yes	–
						ADOS-2							
Gonzalez-Gadea et al. (2015)	Predictive coding	24	19	10.38 (1.97)	11.63 (2.43)	3Di	Raven	39.63 (9.83)	40.16 (8.20)	ND			

All participants have no presence of known genetic condition other than ASD, or major psychiatric disorder (included in inclusion criteria for this systematic review)

*P1*  pre-attentive perceptual processing, *N2* stimulus detection, *P3* stimulus categorization and memory updating, *N400* semantics, *P600* synaptic processing, *MMN* Mismatch negativity is the negative component of a waveform obtained by subtracting event-related potential responses to a frequent stimulus (standard) from those to a rare stimulus (deviant), *SCQ* Social Communication Questionnaire, *ADOS-2* Autism Diagnostic Observation Schedule 2nd Editio, *ADI-R* Autism Diagnostic Interview-Revised, *GARS-2* Gilliam Autism Rating Scale—Second Edition, *KBIT-2* Kaufman Brief Intelligence Test—Second Edition, *DAS-II* Differential Abilities Scale—Second Edition, *BAS-II* British Ability Scales assessment, *ASD/DD* with developmental delay, *ASD/noDD* without developmental delay, *MWT-B* Mehrfachwahl–Wortschatz Intelligenz test, *SNR*  − 16 dB, − 12 dB, − 8 dB noise conditions, *ASD-LI* ASD minus language impairment, *ASD + LI* ASD plus language impairment, *Leiter* Leiter-3 Standard Score, *3Di* Diagnostic and Dimensional Interview, similar to ADI-R, *SRS* Social Responsiveness Scale-2, *SP-2* Sensory Profile-2

*p < 0.05; **p < 0.01 (significant difference); ***p < 0.001 (significant difference)

^#^Amplified 10 dB gain from voice input

~  = diagnose by the Education and Youth Affairs Bureau, which is a governmental and authoritative institution of Macau

Event-related potential waveforms have a series of positive and negative voltage deflections, referred by a letter indicating polarity (N, negative; P, positive) and a number indicating their latency in milliseconds (Luck and Kappenman 2012). As an example, N100 (also known as N1) indicates a negative polarity between 75 and 130 ms, usually associated with pre-attentive perceptual processing followed by P200 associated with stimulus detection (150–275 ms after stimulus presentation) (Key et al. 2005). Other components will be addressed in this review, such as P300 (P3; stimulus categorization and memory updating), N400 (semantics), and P600 (syntactic processing). Mismatch negativity (MMN) is the negative component of a waveform obtained by subtracting event-related potential responses to a frequent stimulus (standard) from those to a rare stimulus (deviant).

Some ASD studies have focused on early ERP components (Table 6), mainly showing reduced P1 latencies and amplitude (Patel et al. 2019; Arnett et al. 2018; Bidet-caulet et al. 2017). Looking particularly to patterns of auditory habituation, data suggest reduced habituation for ASD children in the P1 component and a decrease in MMN amplitude (Ruiz-Martínez et al. 2019). Pronounced habituation slopes have been observed for neurotypical subjects, with greatest difference at channel Fp1 and in the frontal and fronto-central electrodes (Jamal et al. 2020). For neurotypical subjects, P1 is stronger in the initial section of stimuli sequence, showing a gradual reduction in ERP amplitude over time (habituation) (Jamal et al. 2020). On the other hand, the ASD group shows absence of reduction between the first and last ERP, or even a positive slope toward the end of the experiment (reduced habituation) (Jamal et al. 2020). Regarding temporal processing with gap detection tasks, when compared with TD peers, ASD children with intact cognitive skills present reduced P2 amplitude (Foss-feig et al. 2018). The P2 component is associated with attention and stimulus classification, suggesting that the presence of a gap enhances the difficulty of primary level detection, and subsequent perceptual processes may fail to engage. In speech-in-noise tasks, high-functioning ASD adults present higher P2 amplitudes (Borgolte et al. 2021). These results suggest that the P2 component can be affected by the audiovisual process and specificities of both stimuli and condition. Early MMN (around 120 ms) show enlarged responses only for pure-tone context, with no other modulations dependent on action sound context (Piatti et al. 2021). Individual differences in auditory ERPs with four different loudness intensities (50, 60, 70, 80 dB SPL) (Dwyer et al. 2020) were tested using hierarchical clustering analysis based on ERP responses. It was possible to verify a pattern of linear increases in response strength accompanied by a disproportionately strong response to 70 dB stimuli for ASD young children, correlated with auditory distractibility (Dwyer et al. 2020). In a similar study, some clusters of ASD individuals presented weak or absent N2 by 60 dB and increased strength with higher intensities (70 or 80 dB) (Dwyer et al. 2021). However, there was an overlap between ASD and TD participants in several clusters (Dwyer et al. 2020, 2021), suggesting that sensory impairments in ASD might be largely accounted for the wide interindividual variability that exists within the spectrum.

**Table 6:** Summary results of ERP study results, organized by year

Source	Field	Experiment					*n*		Mean age (SD); Range (min–max)		Cognitive standard scores
		Paradigm	Stimuli	dB SPL	Results ASD	ERP results	ASD	TD	ASD	TD	Differences
Borgolte et al. (2021)	Audiovisual speech perception	Temporal dynamics of speech-in-noise	Visual (lip movements) and auditory (voice) speech information that was either superimposed by white noise (condition 1) or not (condition 2)	SNR	Worse speech comprehension noise condition	Higher P2 amplitudes parietal	14	15	40.3 (8.9)	42.4 (12.7)	ND
					No group differences in the NN condition, with a comprehension rate of nearly 100% in both groups						
Chen et al. (2021)	Phonetic encoding	Vowel-speech	Formant-exaggerated speech and non-speech	70	Enhanced P1 for vowel formant exaggeration in the TD group but not in the ASD group	Formant-exaggerated speech: no P1 enhancement as TD	24	24	7.60 (2.32)	7.46 (1.91)	*
					Nonspeech stimuli: similar P1 enhancement in both ASD and TD group						ND
					Differences in neural synchronization in the delta-theta bands for processing acoustic formant changes embedded in non-speech						
Dwyer et al. (2021)	Heterogeneity: individual differences	Listening of tones at four identity levels	Sine waves of complex tones (sine waves of equal amplitude at different frequencies	Varied in intensity (50,60,70,80)	Substantial heterogeneity	Some clusters: weak or absent N2 negativities	243	96	38.50 m (6.02)	37.09 m (6.46)	***
		Hierarchical clustering			Overlap of ASD and TD	Other clusters: N2 responses present at varying latencies					
Piatti et al. (2021)	Attentional orienting to sounds in speech	Passive auditory oddball task	Two deviant stimuli (one vowel sound and one complex tone) either in a speech or in a non- speech context	60	P3a mean voltages, we found an attenuated response in children ASD/noDD when deviant tones were presented in speech, but not in other conditions. Children with ASD/DD did not differ from TD in P3a mean voltages	More negative MMN voltages	ASD/DD: 22	17	ASD/DD: 34.37 (7.11)	38.21 (10.99) m	***
						Attenuated P3a mean voltages ASD/noDD with deviant	ASD/noDD: 12		ASD/noDD: 39.62 (7.54)		
Jamal et al. (2021)	Sensory habituation	Visual and auditory sequences of repeated stimuli	Auditory: beep of 250 Hz; visual: radial checkerboard on a gray background	72	Reduced habituation both auditory and visual stimuli	No reduction between the first and the last ERP	13	22	7.4–12.8	7.1–12.8	ND
					Rates of habituation correlated with several clinical scores	TD: negative slope; SD: positive slope					
Kadlaskar et al. (2021)	Tactile and auditory reactivity patterns	Oddball paradigm	Silent video while being presented with tactile and auditory stimuli	60	Differences in early perceptual processing of auditory (i.e., lower amplitudes at central region of interest), but not tactile, stimuli						
					No differences or later attentional components						
Dwyer et al. (2020)	Heterogeneity: individual differences	Identify subgroups based on the normalized global field power (GFP) of their ERPs to auditory stimuli of four different loudness intensities	Sine waves of complex tones (sine waves of equal amplitude at different frequencies	50, 60, 70, 80	4 clusters; Overlap of ASD and TD	More likely to display a pattern of relatively linear increases in response strength	132	81			
						Disproportionately strong response to 70 dB stimuli					
						Auditory distractibility: disproportionately strong responses to 80 dB					
						Clusters did not differ in the chronological ages of their participants, which suggests that developmental changes in auditory evoked responses do not affect the loudness- dependency of overall response strength in the time-window of the present study					
Green et al. (2020)	Speech sound differentiation	MMN: auditory oddball speech and pure-tone sounds (ASD + LI vs ASD–LI vs TD)	Stimuli matched for frequency, duration and intensity (Praat) + vowel (male speaker)	70	ASD were hypersensitive to sounds	ASD + LI: decreased MMN latency (left hemisphere) in response to novel vowel sound	15	10	ASD–LI: 7.38 (1.19)	0 (1.72)	*
					Increased connectivity in primary sensory cortices at the expense of connectivity to association areas of the brain				ASD + LI: 7.29 (1.70)		***
Knight et al. (2020)	Predictive coding	Predictive coding in rhythmic tone sequences of varying complexity	Repeated five-rhythm tones that varied in the Shannon entropy of the rhythm	70	No differences in the mechanisms of prediction error to auditory rhythms of varied temporal complexity	ASD + TD: decreased MMN	21	19	14	15	*
Schwartz et al. (2020)	Own name	One’s own name (OON) in cocktail scenario	Quiet and multispeaker setting	80	Auditory filtering disruption	TDs and ASD-Vs: significant MMRs to OON multisetting	ASD-V: 27	27	ASD-V: 17.21 (2.08)	17.81 (3.00)	–
					Strength of LPPs positively correlated with auditory filtering abilities		ASD-MLV: 20		ASD-MLV:16.81 (2.64)		
van Laarhoven et al. (2020)	Predictive coding	Prediction errors in auditory prediction by vision	Unexpected auditory omissions in a sequence of audiovisual recordings of a handclap in which the visual motion reliably predicted the onset and content of the sound	61	Unexpected auditory omissions: negative omission response	N1: similar for ASD and TD	29	29	18.64 (2.11)	18.93 (1.22)	ND
DiStefano et al. (2019)	Semantics	Semantic congruence ERP	Pictures were displayed followed by the auditory expected or unexpected word	–	EEG evidence of semantic processing, but it was characterized by delayed speed of processing and limited integration with mental representations	N400 effect with shorter latency in TD	40	18	88.67 (22.04): VASD	91.61 (24.50)	***
						Late negative component present in TD, mid-frontal region in MVASD, not present in VASD			92.42 (22.53): MVASD		***
Grisoni et al. (2019)	Semantic understanding and predictive coding	Biological indicators of sound processing, (action-) semantic understanding and predictive coding	auditory, passive listening, MMN task (sounds: action and non-action words—semantically congruent with regard to the body part they relate to or semantically incongruent or unrelated)	–	Deficits in predictive coding of sounds and words related to action, which is absent for neutral, non-action, sounds	Prediction potential: reduced for action	20	22	38 (10.3)	31.9 (11.1)	ND
					Deficits in semantic processing	Early MMN: enlarged for pure-tone context, no other modulation dependent on action sound context					
Patel et al. (2019)	Prosody	Neural basis of prosodic differences	Pitch-perturbed auditory feedback paradigm during sustained vowel and speech production	70 #	Increased response onset latencies during sustained vowel production	Reduced P1 amplitude	19 (12 M)	20 (12 M)	17.22 (6.30)	14.99 (7.60)	**
Ruiz-Martínez et al. (2019)	Habituation and auditory discriminative process	Electronic and human sounds (standard + deviant)	Human and nonhuman sound Praat + standard tones 415 Hz	65	Lower auditory discrimination	Reduced habituation P1	16	15	8.96 (1.01)	8.86 (1.77)	***
					Increased activation to repeatedly auditory stimulus	Decrease in the amplitude MMN					
Thomas et al. (2019)	Own name	Subject’s own name in preschoolers	Subject’s own name vs. unfamiliar nonsense name	70	Greater negativity to SON over frontal regions	N100 amplitude, SON negativity	19	13	52.29 (9.43)	51.22 (9.05)	***
											***
Zhang et al. (2019)	Non-Speech and Speech Pitch Perception	Lexical tone contrasts and non-speech pitch variations + oddball	Lexical tone (e.g., /ga/)	75	Impaired ability when processing speech pitch information Non-speech: ND	TD: larger MMN responses (speech pitch contour) and stronger MMN (speech pitch height)	16	16	10.42 (2.12)	9.48 (0.86)	ND
						ASD: more positive MMR (speech pitch height)					
Arnett et al. (2018)	Implicit language learning and receptive language ability	Artificial language statistical learning task	Tri-syllabic, artificial, unstressed (i.e., lacking prosodic cues) nonsense word combinations	–	Atypical lateralization of word-learning	TD: attenuated P1 amplitude in the left hemisphere	27	76	12.12 (2.9)	13 (2.3)	**
						ASD: bilateral attenuation					
Charpentier et al. (2018)	Prosody	Prosodic change detection	Vowel /a/ uttered by different female speakers with either neutral or emotional prosody (anger, fear, happiness, surprise, disgust, sadness	70	Change detection altered Differences between children and adults with ASD	Larger P3a amplitude (P3a latencies shorter in adults) Earlier MMN	15	15	10.0 (1.4)	9.8 (1.4)	*
							16	16	26.2 (6.8)	26.2 (6.4)	*
Foss-Feig et al. (2018)	Temporal processing: silence gaps	Electrophysiological response to silent gaps in auditory stimuli	White noise (20 Hz–20 kHz) bursts + silent gaps	80	Degree of P2 amplitude attenuation was highly associated with clinical features, including more prominent sensory symptoms (i.e., auditory processing abnormalities and failure to register sensory input) and weaker processing language skills	Reduced P2 amplitude	15	17	11.86 (1.4)	12.23 (1.2)	ND
Goris et al. (2018)	Sensory Prediction Error	Local prediction error processing is modulated by global context (i.e., global stimulus frequency)	Oddball task: short sequences of either five identical sounds (local standard) or four identical sounds and a fifth deviant sound (local deviant)	70	ASD: less flexible in modulating their local predictions	MMN modulated by global context: smaller effect in ASD	18	24	Adults	Adults	ND
						No differences P3b					
Huang et al. (2018)	Sensitivity to duration contrasts in speech and non-speech contexts	Oddball paradigm	Pure tone + vowel	60	Distinct patterns of discrimination and orienting responses	Pure-tone: diminished response amplitudes and delayed latency MMN; ND P3a	22	20	9.6 (1.88)	9.4 (1.71)	***
						Vowel: smaller P3a;: ND MMN					***
Hudac et al. (2018)	Cognitive response to environmental change	Processing and habituation to deviance sound	Silent video of a trip to the zoo while passively attending to randomly presented frequent tones (70%) + infrequent tones (15%) + novel sounds (15%)	65	Overall heightened sensitivity to change	Greater P3a amplitude to novel sounds	102	31	12.29 (3.56)	13.27 (2.34)	***
		Auditory oddball task				Youth: slower attenuation of the N1 response to infrequent tones and P3a response to novel sounds					***
Lindström et al. (2018)	Prosody	Behavioral sound-discrimination test: natural word stimuli uttered with different emotional connotations	(neutral, sad, scornful and commanding)	56	Anomalous neural prosody discrimination	Differentially distributed on the scalp Diminished amplitude of P3a	15	16	10.4	10.1	ND
					Impaired orienting to prosodic changes						
					Sluggish perceptual prosody discrimination in children with ASD						
Lodhia et al. (2018)	Auditory spatial cues	Spatial cues to auditory object formation – the relative timing and amplitude of sound energy at the left and right ears	Dichotic pitch stimuli – white noise stimuli in which interaural timing or amplitude differences applied to a narrow frequency band of noise typically lead to the perception of a pitch sound that is spatially segregated from the noise	70	ASD: object-related negativity to amplitude cues	P400 missing	15	15	25.80 (6.81)	27.07 (5.80)	ND
					Do not experience a general impairment in auditory object formation						
					Later attention-dependent aspects of auditory object formation missing						
Bidet-Caulet et al. (2017)	Voice perception	Vocal (speech and non-speech) and non-vocal sounds	Vocal and non-vocal sequences	70	ASD: lack of voice-preferential response	TD: voice-sensitive response over right fronto-temporal	16	16	10 y 6 m (1 y 5 m)	10 y 5 m (1 y 5 m)	*
						ASD: atypical response to non-vocal sounds					*
						Smaller P100 non-vocal sounds					
						Smaller right fronto-temporal negative Tb peak non-vocal sounds					
Galilee et al. (2017)	Detection and discrimination of speech and non-speech sounds	Novel paired repetition	Pairs of stimuli (speech sounds, non-speech sounds)	60	Speech versus non-speech detection	N330 match/mismatch responses right hemisphere	14	14	61 m (8.8)	50 m (11)	ND
					Atypical speech versus non-speech processing	Absent effect of match/mismatch 600 ms for non-speech followed by speech					
Wang et al. (2017)	Speech-specific categorical perception	Distinct pitch processing pattern for speech and non-speech stimuli and speech-specific deficit in categorical perception of lexical tones: oddball paradigm	Pitch deviations representing within-category and between-category differences in speech and non-speech contexts	75	Lack of categorical perception in the lexical tone condition	Enhanced within-category MMRs	16	15	10.4	10.3	ND
					Speech-specific categorical perception deficit						
Zhang et al. (2018)	Lexical stress	Oddball paradigm: neural responses bilingual children in L2 lexical stress	MOther (1st syllable stressed) vs. moTHER (deviant)	–	Chinese-English bilingual ASD: less sensitive to lexical stress	Reduced MMN amplitude (left temporal- parietal)	15	16	10.04 (1.53)	9.48 (0.86)	ND
					More negative MMN response for ASD (right central-parietal, temporal-parietal, and temporal sites)						
					ASD: right hemisphere more activated						
Karhson and Golob (2016)	Top-down and bottom-up attentional processes	Oddball target detection	Target, non-target, and distractor	60	TD: top–down control (P3b latency) increased under greater load in controls	ASD: ND P3a	12	13	22.5 (4.1)	22.8 (5.1)	ND
					Enhanced bottom-up processing of sensory stimuli in people with autism	Early ERP responses (P50 amplitude) positively correlated to increased sensory sensitivity					
Key et al. (2016)	Speech sound differentiation	Contrasting consonant–vowel syllables during a passive listening paradigm	Six syllables /ba/, /da/, /ga/, /bu/, /du/, and /gu/	75	Reduced consonant differentiation	84- to 308-ms period	24	18	6. 71 (1.34)	7.14 (1.45)	***
					Related to individual differences in non-verbal versus verbal abilities	ND: 432–700 and 320- to 444-ms interval					
Gonzalez-Gadea et al. (2015)	Predictive coding	Describe mechanisms responsible for attentional abnormalities	Standard and deviant tone sequences (expected and unexpected)	–	Top-down expectation abnormalities could be attributed to a disproportionate reliance (precision) allocated to prior beliefs in ASD	Reduced superior frontal cortex (FC) to unexpected events	24	19	10.38 (1.97)	11.63 (2.43)	ND
					Associated with specific control mechanisms: inhibitory control	Increased dorsolateral prefrontal cortex (PFC) activation to expected events					

All participants have no presence of known genetic condition other than ASD, or major psychiatric disorder (included in inclusion criteria for this systematic review)

*P1* pre-attentive perceptual processing, *N2* stimulus detection, *P3* stimulus categorization and memory updating, *N400* semantics, *P600* synaptic processing, *MMN* mismatch negativity is the negative component of a waveform obtained by subtracting event-related potential responses to a frequent stimulus (standard) from those to a rare stimulus (deviant), *SCQ* Social Communication Questionnaire, *ADOS-2* Autism Diagnostic Observation Schedule 2nd Edition, *ADI-R* Autism Diagnostic Interview-Revised, # amplified 10 dB gain from voice input, *GARS-2*  Gilliam Autism Rating Scale—Second Edition, *KBIT-2* Kaufman Brief Intelligence Test—Second Edition, *DAS-II * Differential Abilities Scale—Second Edition, *BAS-II* British Ability Scales assessment, *ASD/DD* with developmental delay, *ASD/noDD* without developmental delay, *MWT-B* Mehrfachwahl–Wortschatz Intelligenz test, *ASD-LI* ASD minus language impairment, *ASD + LI* ASD plus language impairment, *Leiter* Leiter-3 Standard Score, *3Di* Diagnostic and Dimensional Interview, similar to ADI-R

*P < 0.05; **P < 0.01 (significant difference); ***P < 0.001 (significant difference)

SNR =  − 16 dB, − 12 dB, − 8 dB noise conditions

~  = diagnosed by the Education and Youth Affairs Bureau, which is a governmental and authoritative institution of Macau

A large-scale study tested 133 children and young adults in an auditory oddball task, comparing mismatch negativity and P3a results, as well as temporal patterns of habituation (N1 and P3a) (Hudac et al. 2018). The P3a component is indexed by different levels of attentional orienting. Results showed heightened sensitivity to change to novel sounds in ASD subjects, increased activation upon repeated auditory stimuli, and dynamic ERP differences driven by early sensitivity and prolonged processing (Ruiz-Martínez et al. 2019; Hudac et al. 2018). To test the neural indices of early perceptual and later attentional factors underlying tactile and auditory processing, ASD and age- and non-verbal IQ-matched TD peers were compared (Kadlaskar et al. 2021). Using an oddball paradigm where children watched a silent video while being presented with tactile and auditory stimuli, results showed reduced amplitudes in early ERP responses for auditory stimuli but not tactile stimuli in ASD children. The differences in neural responsivity were associated with social skills (Kadlaskar et al. 2021). Regarding top–down and bottom–up attentional processes, young ASD children with no developmental delay seemed to present more negative MMN voltages and an attenuated response of P3a mean voltages when deviant tones were presented in speech (Piatti et al. 2021). When compared with TD peers, bilingual children with ASD seemed to be less sensitive to lexical stress, with reduced MMN amplitude at the right central–parietal, temporal–parietal, and temporal sites (Zhang et al. 2019). Children with developmental delay did not differ from the control group in the P3a component (Piatti et al. 2021). Compared to adults, there was enhanced bottom–up processing of sensory stimuli in participants with high-functioning autism, with early ERP responses positively correlated to increased sensory sensitivity (Xiaoyue et al. 2017). In ASD children, more positive MMR was observed in the processing of speech pitch height (Zhang et al. 2019), suggesting hypersensitivity to higher frequency tones.

Another relevant feature for auditory processing is prosody, the rhythmic and intonational aspect of language. Event-related potential (ERP) studies show preserved processing of musical cues in ASD individuals, but with prosodic impairments (Depriest et al. 2017). Differences in prosody such as intonation are highly relevant for language-related impairments in ASD with a significant impact on communication. To test prosodic processing, many studies record brain responses to neutral and emotional prosodic deviances reflecting change detection (MMN) and orientation of attention toward change (P3a).

Children with ASD present atypical neural prosody discrimination, with distinct patterns depending on the experimental design. For instance, the P3a component becomes more prominent upon greater difference of stimulus change. Studies using sound-discrimination tasks with lexical tone contrasts based on naturally produced words (e.g., a vowel with neutral or emotional prosody such as sadness) show different P3a depending on the IQ level of ASD children. A study with high-functioning ASD children shows diminished amplitude of P3a (Lindström et al. 2018). In a study comparing children and adults, low-functioning children revealed a larger P3a compared to the control, with P3a latencies shorter in ASD adults (Charpentier et al. 2018). Regarding change detection, ASD adults presented an earlier MMN (Charpentier et al. 2018). Both results suggest atypical neural prosody discrimination and deficits in pre-attentional orientation toward any change in the auditory environment (Lindström et al. 2018; Charpentier et al. 2018). When vowel and pure tone are compared within tone languages (smaller physical difference between standard and deviants compared to non-tone languages), ASD children still presented diminished response amplitudes and delayed latency of MMN for pure tones, and smaller P3a for vowel (Huang et al. 2018). At a pre-attentive perceptual processing level, low-functional ASD children present increased response onset latencies during sustained vowel production, with reduced P1 ERP amplitudes (Patel et al. 2019; Bidet-caulet et al. 2017; Charpentier et al. 2018), lacking neural enhancement in formant-exaggerated speech tasks (Chen et al. 2021). When a non-speech sound is followed by a speech sound, TD children present match/mismatch effects at approximately 600 ms, as opposite to ASD (Galilee et al. 2017). Interestingly, when speech and non-speech sounds are compared between high-functioning ASD children matched on age, gender, and non-verbal IQ with TD group, ASD children show impaired processing ability regarding speech pitch information, but no differences are observed for non-speech sounds (Zhang et al. 2019; Chen et al. 2021). Regarding speech differentiation, there seems to be no differences in voice perception. Low-functioning children with ASD seem to present atypical response to non-vocal sounds (Bidet-caulet et al. 2017), with atypical consonant differentiation in the 84- to 308-ms period, related to individual differences in non-verbal versus verbal abilities (Key et al. 2016).

There is EEG evidence for altered semantic processing in children with ASD, characterized by delayed processing speed and limited integration with mental representations (Piatti et al. 2021; Distefano et al. 2019). Semantic processing refers to encoding the meaning of a word and relating it to similar words or meanings. During passive and active listening, there is a pre-attentive stage of sound segregation. When individuals attend to auditory stimuli, there are some components that can distinguish the stage of neural processes. The Subject’s own name (SON) is a unique auditory stimulus for triggering an orienting response. Sounds need to stand out from the background to elicit an orienting response. SON is automatically distinguished from other names without reaching awareness, causing involuntary attention take (Tateuchi et al. 2015). Children with ASD present the ability to selectively respond to one’s own name with greater negativity over frontal regions, reflecting early automatic pre-attentive detection (Thomas et al. 2019), when compared with TD peers. After the pre-attentional level, there is an orienting response to cause a shift of attention, where the auditory system makes use of amplitude and timing cues to differentiate sounds from different spatial locations, giving them context. Although high-functioning adults with ASD do not seem to have general impairment in auditory object formation, they seem to display alterations in attention-dependent aspects of auditory object formation (P400 missing) (Thomas et al. 2019), as well as disruption of auditory filtering (Huang et al. 2018). These deviations at top–down processing might be linked with deficits in perceptual decision-making.

Some studies highlight the importance of proper ability to anticipate upcoming sensory stimuli. ASD adults seem to be less flexible than controls in the modulation of their local predictions (Goris et al. 2018), affecting their function of global top–down expectations. Adults diagnosed with ASD present deficits in predictive coding of sounds and words in action–sound context, with no deficits in predictive coding for neutral, non-action, and sounds (Grisoni et al. 2019; Laarhoven et al. 2020). When using varied auditory rhythms, both ASD and TD presented decreased MMN, with no difference in error prediction (Knight et al. 2020).

There are some interesting data regarding cognitive function lateralization in children with ASD. When comparing ERP responses for speech and non-speech sounds, speech-related events where only detected over temporal electrodes in the left hemisphere (Galilee et al. 2017), compared to bilaterally N330 match/mismatch responses in neurotypical children (Galilee et al. 2017). Regarding ASD children with impairments specifically at language level, results show hypersensibility to sounds with decreased MMN latency at the left hemisphere (Green et al. 2020). Older children with non-verbal IQ impairments presented bilateral attenuation in a word-learning task, as opposed to attenuated P1 amplitude present in the left hemisphere of TD children (Arnett et al. 2018). Young children with high-functioning ASD seem to fail to activate right-hemisphere cognitive mechanisms, probably associated with social or emotional features of speech detection.

#### Increased auditory perceptual capacity: impact on social and emotion perception

Impairments at the level of emotional perception can be related to internal distractions or overload of sensory information that hinder social communication. Studies using sympathetic skin response show that children diagnosed with ASD exhibit delayed habituation to auditory stimuli (Bharath et al. 2021). The predominant state of sympathetic nerves can affect the predisposition to filter and perceive cues, and also influence anxiety levels or valence of social cues. Individuals with ASD seem to present higher levels of perceptual capacity, which might be correlated with higher levels of sensory sensitivity (processing more information at any one time) (Brinkert and Remington 2020).

Emotional expressiveness is highly important for emotional perception, carrying non-verbal information that helps forming social judgments, and predisposing individuals for further social engagement. A study focusing on emotion recognition in intellectually disabled children with ASD found that these children displayed poorer performance in recognizing surprise and anger in comparison to happiness and sadness (Golan et al. 2018). A different study tested emotion performance by comparing ASD children with siblings without ASD diagnosis and TD peers (Waddington et al. 2018). Interestingly, the authors found not only poorer emotion performance in terms of speed and accuracy in the ASD group, but also poorer performance in the ASD sibling group compared to TD controls (Waddington et al. 2018), suggesting a possible contextual influence in emotional perception.

Children with ASD seem to present lower autonomic reactivity to human voice, with impairments in the vocal emotion recognition tests, albeit normal pro-social functioning (social awareness and social motivation) (Anna et al. 2015; Schelinski and Kriegstein 2019). This interesting result raises the question whether social impairments in ASD could be a consequence of hyperarousal from sensory overload.

Regarding other assessment paradigms, children with ASD seem to struggle with face-to-face matching, when compared to voice-face and word-face combinations (Golan et al. 2018), with worst performance in noisy environments (Newman et al. 2021). The ability to integrate facial-voice cues seems to be correlated with socialization skills in children with ASD (Golan et al. 2018).

Together, these studies highlight the importance of reappraising cognitive function in light of the sensory systems, such as the auditory system: the Peripheral Auditory System, where auditory pathway starts, as well as the Central Auditory Nervous System, where all auditory information gets integrated and processed.

### Assessing the integrity of peripheral auditory system

Sounds are produced by acoustic waves that reach the external auditory canal and travel to the eardrum causing vibration of the tympanic membrane at specific frequencies (the typical hearing frequency range in humans is 20 to 20.000 Hertz, cycles per second) (Peterson et al. 2022). The vibration of the tympanic membrane causes vibration of tiny bones in the middle ear that amplify the signal and send it to the cochlea. The signal travels to fluid-filled sections of the cochlea, the scala vestibuli and the scala tympani, and oscillations of these sections transmit energy to the scala media, causing shifts between the tectorial and basilar membrane. The basilar membrane contains receptor hair cells that can be either activated or deactivated by shifts that open or close potassium channels. Cells near the base of the cochlea respond to high frequencies, with increased flexibility to respond to lower frequencies toward the apex of the cochlea (Peterson et al. 2022; Zhao and Müller 2015; Delacroix and Malgrange 2015). Inner hair cells are responsible for the majority of auditory processing. Outer hair cells synapse only on 10% of the spiral ganglion neurons (Delacroix and Malgrange 2015). Neurons within the spiral ganglion mostly synapse at the base of hair cells to the auditory nerve (cochlear nerve). The cochlear nerve then sends up information to the brain cortex through a serious of nuclei in the brainstem: the cochlear nuclei (medulla), superior olivary complex (pons), lateral lemniscus (pons), inferior colliculus (midbrain), and medial geniculate nucleus (midbrain) (Felix et al. 2018). Although the primary auditory pathway mostly ascends to the cortex through the contralateral side of the brainstem, all levels of the auditory system have crossing fibers, receiving and processing information from both the ipsilateral and contralateral sides (Peterson et al. 2022).

#### The auditory brainstem response (ABR) in ASD

Reported cognitive deficits in ASD regarding speed and accuracy of sound stimuli assessment (Distefano et al. 2019; Waddington et al. 2018) might potentially be contributed by impairments in impulse initiation at the cochlear nerve, or impairments in the transmission and conduction of signals along the brainstem, as it occurs in demyelinating diseases. One tool used to measure neural functionality of the auditory brainstem is the auditory brainstem response (ABR) (Celesia 2015; Jewett and Williston 1971). Participants usually perform hearing screenings to exclude hearing disabilities, such as lesions below or within the cochlear nuclei. Of note, since 2015, there are few experimental studies assessing the integrity of the peripheral auditory system in ASD.

The auditory brainstem response (ABR) measures electrical signals associated with the propagation of sound information through the auditory nerve to higher auditory centers, after an acoustic stimulus. Major alterations in neuronal firing along this pathway can be detected as changes in auditory brain response ([99]). Complementary to the use of a short click as a classical acoustic stimulus, ABR is also often performed with complex sounds, such as syllables, which incorporate an array of complexity more similar to speech. Two major categories of ABR stimulus are detailed below: click-ABR and speech-ABR.

Most ABR studies report differences in auditory brainstem processing for ASD individuals when compared with control groups (Table 7). Results are discussed taking into consideration the age of participants and different time-points, their cognitive pattern, type of stimuli, and experimental design.

**Table 7:** Summary of publications sources used in this systematic review, related with ABR, organized by year

Source	Stimuli	Country	*n*		Mean age (SD)		ASD confirmation tools	Timepoints
			ASD	TD	ASD	TD		
Delgado et al. (2021)	C	USA	370 (286 M)	128,181 (63882 M)	1.74 (2.93) d	1.80 (2.98) d	DSM-V	2
Fujihira et al. (2021)	C	Japan	17 (15 M)	20 (17 M)	30.5 (4.7)	29.3 (3.9)	DSM-V	1
							**IQ > 85**	
ElMoazen et al. (2020)	C	Egypt	20 (16 M)	20 (16 M)	4.99 (2.59)	5.02 (2.64)	DSM-V	1
Jones et al. (2020)	C + S	USA	18 (13 M)	18 (13 M)	2.941 (0.45) y	3.058 (0.35) y	ADOS	1
Tecoulesco et al. (2020)	C + S	USA	12 (11 M)	12 (10 M)	19.90 (1.20) m	30.93 (5.87) m	ADOS	2
Claesdotter-Knutsson et al. (2019)	C	Sweden	39 (18 M)	34 (23 M)	11.50 (3.0) y (M)	13.18 (3.2) y (M)	ADOS	1
					12.71 (3.36) y (F)	13.12 (3.47) y (F)		
Chen et al. (2019)	S	China	15 (12 M)	20 (14 M)	4.86 (1.48) y	4.57 (0.53) y	GDDS	2
							CARS	
Ramezani et al. (2019)	S	Iran	28 (28 M)	28 (28 M)	14.36 (1.86) y	14.99 (1.92) y	DSM-V	1
							**IQ > 85**	

*S* speech-ABR, *C* click-ABR, *M* male, *ASD* autism spectrum disorder, *TD* typical development, *y* years, *m* months, *d* days, *F* female, *M* male

##### Click-evoked brainstem responses (click-ABR) in ASD

In the first 10 ms after a click, click-ABR produce five-to-seven waveforms (wave I–VII). These wave peaks reflect the propagation of electrical activity as it travels along the auditory pathway, providing information in terms of latency (speed of transmission), amplitude of the peaks (interpreted as the number of neurons firing), inter-peak latency (time between peaks), and interaural latency (correlation between left and right ear) (Musiek and Lee 1995).

Some studies with click-ABR (Table 8) show longer latencies for ASD participants in wave V, and longer latency of inter-peak intervals in waves I–V and III–V (Tecoulesco et al. 2020; Jones et al. 2020). However, these experiments were done with toddlers. Interestingly, a recent study tested older children in a click-ABR paradigm (Claesdotter-Knutsson et al. 2019). Results do not show differences in ABR latency, but reveal a higher amplitude of wave III in ASD, suggesting functional alterations at the pons region (Claesdotter-Knutsson et al. 2019). This experiment used binaural sound exposure, showing a higher degree of correlation between left and right ear in the ASD group. A different study found absence of asymmetry in the latency of wave V between the right and left sides, both in ASD and control groups (ElMoazen et al. 2020). The authors further report a reduced amplitude in the binaural interaction component in younger children with ASD, which might reflect reduced binaural interaction at younger ages not related to artificial latency shift (ElMoazen et al. 2020). A recent study looking at newborns later diagnosed with ASD at the age of 3–5 years showed ABR latency delays (Delgado et al. 2021), suggesting the emergence of differences in acoustic processing, at the brainstem level, right after birth. A more recent study tested adults with ASD and found no differences in absolute ABR wave latencies (Fujihira et al. 2021). The authors showed a shorter summating potential (SP), suggesting normal auditory processing in the brainstem for ASD adults (Fujihira et al. 2021).

**Table 8:** Summary results using click-ABR and speech-ABR, organized by year

Source	Stimuli	dB SPL	Material	Sound	Latency	Amplitude	Other measures
					ASD	ASD	ASD
Delgado et al. (2021)	Click	35 nHL	Earphones	Right, left	Delayed	NF	T1: greater newborn ABR phase values
							T2: slower neurological responses
Fujihira et al. (2021)	Click	100 (67.9 nHL)	Earphones	Right ear	Shorter SP	NF	
ElMoazen et al. (2020)	Click	65 nHL	Headphones	Right, Left	longer BIC	Reduced BIC	No asymmetry in the latency of wave V between the right and left side in both groups
				Binaurally	*Delay at wave V		
Jones et al. (2020)	Click	98.5	Earphones	Right ear	Longer interpeaks waves I–V; III–V	ND	No differences between auditory processing and behavioral measures
	Syllable /da/	80			Higher in wave O		
Tecoulesco et al. (2020)	Click	80	Earphones	Right ear	Longer wave V	ND	Stability of encoding: similar developmental patterns
	Syllable /da/	80			ND		
Claesdotter-Knutsson et al. (2019)	Click	80	Headphones	Binaurally	ND	Higher wave III	Higher degree of correlation between left and right ear
Chen et al. (2019)	Syllable /da/	80	Headphones	Right ear	T1: prolonged in wave V, A	T1: smaller in wave E	Positive correlation between wave A amplitude and GDDS language score
					T2: prolonged in wave F	T2: prolonged in wave F	
					T1—> T2: shorted wave V	T1—> T2: increased wave A, C	
Ramezani et al. (2019)	Syllable /da/	80	Earphones	Right ear	Longer in wave V, A, D, E, F	ND	Shorter SNR
					Longer V-A		Longer RMS amplitude

Note: only wave *V* was detected in newborns

*dB SPL* decibel sound pressure level, *T1* timepoint1, *T2* timepoint2, *ND* no differences, *SNR* obtained from the mean amplitude of the response divided by the mean amplitude of the pre-stimulus activity, *SP* summating potential, *NF* not referred, *BIC* binaural interaction component

##### Speech-evoked brainstem responses (speech-ABR) in ASD

The most common speech-ABR stimulus used is the universal syllable/da/. After the stimulus, a subcortical response emerges as an ABR waveform of seven peaks (V, A, C, D, E, F, O). Peaks can reflect either a change in the stimulus (i.e., onset, offset, or transition) or the periodicity of the stimulus. There are two main components in speech-ABR: the onset response (waves V and A) and the frequency-following response (FFR; waves D, E, and F). The wave C represents the consonant–vowel transition, while the wave O represents the end of the vowel. Analysis of FFR includes measurements of response timing (peaks), magnitude (robustness of encoding of specific frequencies), and fidelity (comparison of FFR consistency across sessions, which gives an index of how stable the FFR is from trial to trial (Krizman and Kraus 2019)).

Similarly to click-ABR, speech-ABR studies (Table 8) also show longer latencies for ASD participants, including high-functioning ASD individuals (Ramezani et al. 2019). Recently, a longitudinal study included two assessment time-points (interval of 9.68 months) to look for longitudinal changes in speech-ABR (Chen et al. 2019). No differences were found in the TD group, whereas differences were found in the ASD group (shorter latency wave V and increased amplitude wave A and C), suggesting an age effect for ASD (Chen et al. 2019). Another study had two assessment time-points to investigate neural response stability, a metric measured by trial-by-trial consistency in the neural encoding of acoustic stimuli (Tecoulesco et al. 2020). Results showed that children with a more stable neural encoding of speech sounds, in both groups ASD and TD, demonstrated better language processing at a phonetic discrimination task (Tecoulesco et al. 2020). Individuals’ performance was assessed in a different study through listening structured and repetitive listening exercises, with increasing difficulty levels (Ramezani et al. 2021). Results showed gradual improvement in ASD individuals’ temporal auditory skills (Ramezani et al. 2021).

A relevant association has been found between sensory overload and behavioral measures in ASD, but without relevant auditory processing association (Font-Alaminos et al. 2020). For children with ASD, the FFR signal has been described as unstable across trials (Otto-Meyer et al. 2018), tending to increase with stimulus repetition (Font-Alaminos et al. 2020), which suggests an unstable neural tracking at the level of subcortical auditory system (Table 9). The development pattern of auditory information processing was assessed in preschool children with ASD (Chen et al. 2019). Results show a positive correlation between wave A and Gesell Developmental Diagnosis Schedules (GDDS) language score (Chen et al. 2019). According to authors, the latency between V and A complex could suggest a weakened synchronization of neural response at the beginning of speech stimulus (Chen et al. 2019). A different study also revealed longer latencies of the transient FFR components (which include D, E, and F, ABR waves) in the ASD group (Ramezani et al. 2019). These results suggest a possible disturbance in brain pathways implicated in FFR generation [the direct pathway to the contra-lateral IC via the lateral lemniscus, and the ipsilateral pathway via superior olivary complex and the lateral lemniscus (Ramezani et al. 2019)] further raising the question whether this could be a potential compensatory mechanism in ASD.

**Table 9:** Summary results testing FFR, organized by year

Source	Stimuli	dB SPL	Study	Sound	Results	*n*		Mean age (SD)		Cognitive standard scores
					ASD	ASD	TD	ASD	TD	Mean (SD)
Font-Alaminos et al. (2020)	AM pure tones	75	Ability to filter out auditory repeated information	Right	Increase of FFR with stimulus repetition	17	18	9.1 (1.7)	8.8 (1.9)	IQ ≥ 100
	Roving-frequency paradigm				Related with sensory overload					
Otto-Meyer et al. (2018)	Click + syllable	60–80	Neural stability in response to sound	Right	Less stable FFRs to speech sounds reduced auditory stability	12	12	10.71 (2.07)		IQ ≥ 80
	Voiced with a flat pitch									

*AM* Amplitude-modulated

#### Otoacoustic emission (OAE) studies in ASD

The integrity of the peripheral auditory system can also be evaluated using otoacoustic emissions (OAEs). Evoked otoacoustic emissions are produced by healthy ears in response to an acoustic stimulus delivered into a sealed ear canal. The acoustic stimulus causes basilar membrane motion which triggers an electromechanical amplification process by the cochlear outer hair cells, producing a sound that echoes back into the middle ear (otoacoustic emissions). These nearly inaudible emissions are measured using a sensitive microphone and help evaluate normal cochlear function. A study tested a group of children and adolescents with ASD (ASD with Full-Scale IQs higher than 85) with normal audiometric thresholds (Bennetto et al. 2016). Results showed that children with ASD presented reduced OAEs at 1 kHz frequency range, with no differences outside this critical range (at 0.5 and 4–8 kHz regions) (Bennetto et al. 2016), thus suggesting reduced outer hair cell function at 1 kHz. Outer hair cells synapse directly with neurons originating in the nuclei of the superior olivary complex. A morphological post-mortem study of subjects with ASD showed significantly fewer neurons in the Medial Superior Olive (MSO), a specialized nucleus from the superior olivary complex (Mansour and Kulesza 2020). Results also showed that the existing fewer neurons in the MSO are smaller, rounder, and with abnormal dendritic orientations (Mansour and Kulesza 2020). However, the small sample size, variability of post-mortem tissue origin and quality (7 post-mortem samples from drowning, seizure, or other death causes), may hinder conclusions from this study. Previously, a different study found no evidence regarding asymmetrical or reduced middle ear muscle (MEM) reflexes and binaural efferent suppression of transient evoked otoacoustic emissions responses (Bennetto et al. 2016). However, a more recent study showed OAE asymmetry, with the medial olivocochlear system being apparently more effective in the right than the left ear in ASD (Aslan et al. 2022).

### Assessing the integrity of central auditory nervous system

ASD is a neurodevelopmental disorder with behavior and cognitive traits associated with atypicalities in the central nervous system (CNS). Integrity of the central nervous system can be assessed experimentally by several techniques, such as magnetoencephalography (MEG; Table 10), magnetic resonance imaging (MRI; Table 11), as well as other multimodal tools (Table 12).

**Table 10:** Summary of publications sources using MEG, organized by year

Experiment							*n*					Mean age (SD)			
Source	Method	Study	Paradigm	Stimuli/measures	Results	Cognitive standard scores						*or *Mean age (min range- max range)			
						Relevant results	ASD			TD		ASD			TD
Yoshimura et al.(2021)	MEG	P1m	Bilateral auditory cortical response (P1m)	Sinusoidal pure tones	ASD: shorter P1m latency in the left hemisphere	Mental Processing Scale: TD > ASD ***	29			46		74.7 (10.8) m			70.3 (5.9) m
					Correlation between P1m latency and language conceptual ability										
Matsuzaki et al. (2020)	MEG	M50 and M100: comparison between children and adolescents	Signals recorded: left and right superior temporal gyrus	Auditory presentation of tones	ASD: Delayed M50 and M100 latencies	ND: IQ (mean) > 100	58 children			36 children		10.07 (2.38)			9.21 (1.6)
					Differences in M50 and M100 persisted in adulthood	ND: IQ (mean) > 100	19 adolescents			19 adolescents		23.80 (6.26)			26.97 (1.29)
Ono et al. (2020)	MEG	ASSR	Assessing neural synchrony at specific response frequencies	ASSR at 20 Hz and 40 Hz	ASD + TD: Responses to 20 Hz and 40 Hz detected	TD > ASD	23			32		74.8 (11.2) m			69.7 (6.2) m
					ASD + TD: right dominance of the 40-Hz ASSR										
					TD: right-side 40-Hz ASSR was correlated with age										
Seymour et al. (2020)	MEG	ASSRs + tGBR	Replicate and extend findings regarding reductions in ASSRs at 40 Hz	1.5 s-long auditory clicktrain stimulus	ASSRs: bilateral primary auditory regions	ND: Raven score >40	18			18		16.67 (3.2)			16.89 (2.8)
					ND for tGBR from 0-0.1 s following stimulus										
					ASD: reduced oscillatory power at 40 Hz from 0.5 to 1.5 s post-stimulus onset, for both left and right A1										
					ASD: reduced inter-trial coherence (phase consistency over trials) at 40 Hz from 0.64-0.82 s for right A1 and 1.04-1.22 s for left A1										
Stroganova et al. (2020)	MEG	Pitch processing	Spectrally complex periodic sounds (ASSR + SF)	Investigate the ASSR and SF evoked by monaural 40 Hz click trains	SF and ASSR: dominated in the right hemisphere	ASD < TD ***	35			37		9.69 (1.5)			10.08 (1.5)
					SF and ASSR: higher in the hemisphere contralateral to the stimulated ear										
					ASSR: ASSR increased with age both groups										
					SF: moderately attenuated in both hemispheres ASD										
					SF: markedly delayed and displaced in the left hemisphere (ASD boys)										
Wagley et al. (2020)	MEG	Predictive processing with naturalistic statistical learning task	Speech segmentation: neural signals of statistical learning	Evoked neural responses to syllable sequences in a naturalistic statistical learning corpus - left primary auditory cortex, pSTG, IFG, across three repetitions of the passage	TD: neural index of learning in all three ROIs measured	IQ TD > (ASD) > 90 **	15			14		10.06 (1.47)			10.00 (1.64)
					TD: change in evoked response amplitude as a function of syllable surprisal across passage repetitions										
					TD: surprisal increased -> amplitude of the neural response increased (after repeated exposure)										
					ASD: did not show this pattern of learning										
Matsuzaki et al. (2019a)	MEG	MMF	MMF delays in extremely language impaired ASD	MFF responses bilaterally during an auditory oddball paradigm with vowel stimuli	ASD-MVNV: bilaterally delayed MMF latencies	CELF CLI >85; “ASD-V“	ASD-V	ASD-LI	ASD-MVNV	27		ASD-V	ASD-LI	ASD-MVNV	
					delayed MMF responses associated with diminished language and communication skills	CELF core language index <85; “ASD-LI”	27	21	9			10.55 (1.21)	10.67 (1.21)	9.67 (1.41)	10.14 (1.38)
					TD: leftward lateralization of MMF amplitude										
					ASD-MVNV and verbal ASD: abnormal rightward lateralization										
Roberts et al. (2019)	MEG	M50 and M100: study with ASD-MVNV and ASD-V	Signals recorded: left and right superior temporal gyri	Tone stimuli	ASD-MVNV: delayed M50 and M100 latencies, greater than ASD-V	Full-Scale IQ: TD > ASD-V***	ASD-V		ASD-MVNV	34		ASD-V		ASD-MVNV	10.18 (1.36)
					Latencies were associated with language and communication skills	Non-verbal IQ: TD > ASD-V > ASD-MVNV***	55		16			10.64 (1.31)		9.85 (1.32)	
Brennan et al. (2019)	MEG	Predictive processing with naturalistic language	Predictive sentence comprehension during story-listening	Listen to an audiobook story	Predictive parsing equivalent between high-functioning individuals with ASD and TD peers	ND: IQ (mean) > 100	14			13		9.4			9.8
					Neuromagnetic signals correlated with word-by-word states	VERBAL: TD > ASD***									
					Linguistic prediction in school-aged children										
Matsuzaki et al. (2019b)	MEG	MMF	MMF and auditory language discrimination of vowel stimuli	Auditory oddball paradigm with vowel stimuli (/a/ and /u/).	ASD: MMF delayed	ND: IQ (mean) > 100	9			16		22.22 (5.74)			27.25 (6.63)
					ASD: earlier M100 component to single stimulus tokens delayed										
					No correlation between delayed M100 and MMF										
					TD: leftward lateralization of MMF amplitude										
					ASD: rightward lateralization MMF amplitude										
Lambrechts et al. (2017)	MEG	Interval timing	Processing of duration as compared to pitch	Comparison of two consecutive tones according to their duration or pitch	ASD: less able to predict the duration of the standard tone accurately	ND: IQ (mean) > 100	18			18			25:3 (8:1)		25:3 (8:1)
					Engage less resources for the Duration task than for the Pitch task regardless of the context								y:m		y:m
					Lower sensitivity for duration discrimination behaviourally in ASD										
					ASD adults are less able to predict the offset of a standard tone										
Demopoulos et al. (2017)	MEG	Auditory and Somatosensory Cortical Responses	Indices of auditory and somatosensory cortical processing	Magnitude of responses to both auditory and tactile stimulation	ASD: delayed M200 latency response from the left auditory cortex	IQ (mean) TD > ASD > 100**	18			19		9.82 (1.17)			9.79 (1.11)
					ASD: delayed somatosensory response										
					ASD: left M200 latency delay was significantly associated with performance on the WISC-IV Verbal Comprehension Index										
					Cortical auditory response delays were not associated with somatosensory cortical response delays or cognitive processing speed										
Mamashli et al. (2017)	MEG	Auditory Processing in Noise	Cortical responses with passive mismatch paradigm.	Paradigm 1) in a quiet background, 2) in the presence of background noise	Quiet condition: common neural sources of the MMF response in both groups (RTG + IFG)	Verbal IQ: ND	19			17		13 (3)			12 (2)
			Temporal and frontal cortical locations, and functional connectivity with spectral specificity between those locations		Noise condition: MMF response in the right IFG was preserved in the TD group, but reduced relative to the quiet condition in ASD group	Non-verbal IQ: TD>ASD*									
					Noise: reduced normalized coherence in the beta band (14–25 Hz) between left temporal and left inferior frontal sub-regions										
					Unnormalized coherence significantly increased in ASD in multiple frequency bands										
Matsuzaki et al. (2017)	MEG	MMF	MMF and M100: children with ASD who experience abnormal auditory sensitivity	Auditory oddball paradigm (standard tones: 300 Hz, deviant tones: 700 Hz)	ASD_S : longer temporal and frontal residual M100/MMF latencies	ND: IQ (mean) > 100	ASD_S	ASD_noS		TD		ASD_S	ASD_noS		TD
					Prolonged residual M100/MMF latencies were correlated with the severity of abnormal auditory sensitivity in temporal and frontal areas of both hemispheres		11	9		13		9.62 (1.82)	9.07 (1.31)		9.45 (1.51)
Yoshimura et al. (2017)	MEG	MMF	Presence of a speech onset delay (ASD - SOD and ASD - NoSOD)	Oddball sequences: standard stimuli (456 times, 83%) and deviant stimuli (90 times, 17%)	ASD: decreased activation in the left superior temporal gyrus (MMF amplitude)	Mental Processing Scale	ASD-SOD		ASD - NoSOD	46		ASD-SOD		ASD - NoSOD	58.4 (37–79)
					ASD: significant negative correlation between the MMF amplitude in the left pars orbitalis and language performance	ASD-SOD < ASD-NoSOD	23		24			58.1 (40–72)		62.5 (40–72)	
					ASD - SOD: exhibited increased activity in the left frontal cortex (i.e., pars orbitalis)	ASD-SOD < TD									
						Vocabulary TD>ASD									
Brennan et al. (2016)	MEG	Receptive language: cascading effects on speech sound processing	Beamformer source analysis was used to isolate evoked responses (0.1–30 Hz) to stimuli in the left and the right auditory cortex	Nonce linguistic stimuli that either did or did not conform to the phonological rules that govern consonant sequences in English	Phonological processing is impacted in ASD	IQ TD > (ASD) > 90**	12			13		9.3 (1.4)			9.7 (1.4)
					Right auditory responses: attenuated response to illegal sequences relative to legal sequences that emerged around 330 ms after the onset of the critical phoneme										
Ganesan et al. (2016)	MEG	Cortical auditory evoked responses	Somatosensory domain in rapid processing of tactile pulses	Sequence of two tactile pulses with different (short and long) temporal separation	No group difference in the evoked response to pulses with long (700 ms) temporal separation	ND: verbal, and non-verbal IQ > 100	12			22		12.5 (5.21)			13.77 (3.72)
					No group differences in the evoked responses to the sequence with a short (200 ms) temporal separation	Touch score TD > ASD**									
Kurita et al. (2016)	MEG	AEF synchronization	Global coordination across spatially distributed brain regions using Omega complexity analysis - global coordination of AEFs		ASD: higher Omega complexities time-window 0–50ms	ND: IQ (mean) > 90	50			50		66.7 (38 – 92)			66.8 (36 – 97)
					Lower right-left hemispheric synchronization							m			m
Port el al. (2016)	MEG	Auditory response maturation	Longitudinal study: bilateral primary/secondary auditory cortex time-domain (100 ms evoked response latency (M100)) and spectrotemporal measures (gamma-band power and inter-trial coherence (ITC))	Sinusoidal pure tones	ASD_1 + ASD_2: M100 latencies delayed, associated with clinical ASD severity	ND Full IQ >100	ASD	"had ASD"		9	Timepoint 1	8.4 (1.1)	8.7 (0.7)		8.4 (1.3)
					ASD: gamma-band evoked power and ITC reduced	Verbal Comprehension TD>ASD*	22	5			Timepoint 2	12.1 (1.3)	11.8 (0.4)		11.9 (1.5)
					ND: M100 latency and gamma-band maturation rates "had-ASD": exhibited M100 latency and gamma-band activity mean values in-between TD and ASD at both time-points										
Yau et al. (2016)	MEG	Speech and non-speech processing	Association between poor spoken language and atypical event-related field (ERF) responses	Speech and non-speech sounds	ASD: poor spoken language scores associated with atypical left hemisphere brain responses (200 to 400 ms) to both speech and non-speech	IQ TD > (ASD)**	14			18		10.81 (1.71)			10.02 (2.39)
Edgar et al. (2015)	MEG	Neuromagnetic Oscillations phenomena	Test oscillatory phenomena in ASD in terms of frequency and time (STG auditory areas)	Pure tones at 200, 300, 500, and 1,000 Hz	ASD: pre-stimulus abnormalities across multiple frequencies	ND IQ > 100	105			36		10.07 (2.37)			10.90 (2.78)
					Early high-frequency abnormalities followed by low-frequency abnormalities	Core language TD > ASD**									
Gandhi et al. (2015)	GSR, MEG	Auditory habituation	Autonomic and electrophysiological evidence: sensitivity and habituation	1st study: GSR (beep presentation)	Consistent patterns of reduced habituation in ASD	No IQ measures obtained	13			13		27.1 (5.9)			28.9 (5.1)
					GSR_TD: predicted steady decline consistent with habituation										
					GSR_ASD: no decline + steady increase in the GSR over the course of the session										
				2nd study: MEG (early vs late responses)	MEG_TD: early ERFs stronger							15.12 (5.6)			14.75 (5.9)
					MEG_ASD: unchanged amplitude ERFs over time										

*ASD-MVNV* minimally verbal or non-verbal children who have ASD, *ASD-V* verbal individuals who have ASD and no intellectual disability, *IFG*  inferior frontal gyrus, *RTG* right temporal gyrus, *STG* superior temporal gyrus, *LI* language impairment, *AEF* auditory evoked field, *ERFs* sensory evoked response fields, *ASSR* auditory steady-state response, *SF* sustained field, *SS* defined based on the CELF-4 core language index percentile, *ASD-SOD* presence of a speech onset delay, *pSTG* posterior superior temporal gyrus, *ROIs* regions of interest, *GSR* galvanic skin response, *ASD_S* with abnormal auditory sensitivity, *ASD_noS* without abnormal auditory sensitivity, *MRS* magnetic resonance spectroscopy, *Gamma* gamma-band activity

Surprisal = quantify how much information a particular word contributes given some linguistic context. Unexpected words contribute more information—they have high surprisal—as compared to highly expected words

"had-ASD" = subjects with ASD at timepoint 1, and not at timepoint 2

***Significantly different from TD at *p* < 0.001; **Significantly different from TD at *p* < 0.01; *Significantly different from TD at *p* < 0.05

**Table 11:** Summary of publications sources using MRI, organized by year

Source	Field	Method	Experiment				*n*			Mean age (SD)	
			Paradigm	Task/stimuli	Results	Cognitive standard scores Relevant results	ASD		TD	*or *Mean age (min range–max range)	
										ASD	TD
Charpentier et al. (2020)	Brain hemodynamic	fMRI	Oddball paradigm	Brain responses to vocal changes with different levels of saliency (deviancy or novelty) and different emotional content (neutral, angry).	Brain processing of voice and deviancy/novelty appears typical in adults with ASD	ND performance IQ	14		16	27.9 (6.4)	26.4 (7.5)
					No group difference between control and ASD was reported for vocal stimuli processing or for deviancy/novelty processing, regardless of emotional content	Verbal IQ TD > ASD*					
Murray et al. (2020)	Cortical neural inhibition	fMRI	Disrupted cortical neural inhibition and neural responses	Comparing fMRI response magnitudes to simultaneous visual, auditory, and motor stimulation	ASD: No increases in the initial transient response in any brain region - there is no increase in overall cortical neural excitability	ND: mean IQ > 100	18		32	23	24
					ASD: widespread fMRI magnitude increases in response following stimulation offset, approximately 6–8 s after the termination of sensory and motor stimulation						
					ASD: higher fMRI offset - attributed to a lack of an “undershoot”						
					TD: Offset response magnitude associated with reaction times (RT)						
					ASD: overall reduced RT						
Raatikainen et al. (2020)	Whole-brain dynamics	3D magnetic resonance encephalography	Whole-brain dynamic lag pattern variation	Resting-state networks (RSNs)	10.8% of the 120 RSN pairs had statistically significant dynamic lag pattern differences that survived correction with surrogate data thresholding	ND: mean GAI > 100	20		20	23.7 (3.2)	25.3 (6.2)
			Novel technique called dynamic lag analysis (DLA)		Alterations in lag patterns: salience, executive, visual, and default-mode networks						
					92.3% of the significant RSN pairs : shorter mean and median temporal lags in ASD (84.6% TD)						
Pegado et al. (2020)	Temporal voice area	fMRI	The “population thinking”: audio-visual ‘social norm inference’ task	Imagine how most people would judge the appropriateness of vocal utterances in relation to different emotional visual contexts	ASD: more interindividual variability in these judgments despite equal within-participant reliability	ND: mean IQ > 100	22		22	22.5 (4.09)	22.8 (2.94)
				Watching a visual display and hearing auditory input (vocal reaction)	ASD + TD: similar neural representations						
					ASD: more interindividual variability at TVA						
					Larger neural idiosyncrasy in a high-level auditory area - larger behavioral idiosyncrasy (judging auditory valence)						
Abrams et al. (2019)	Voice processing	fMRI	Social communication abilities and activation in key structures of reward and salience processing regions	Neural responses elicited by unfamiliar voices and mother’s voice	ASD: Functional connectivity between voice-selective and reward regions during voice processing predicted social communication	ND full-scale IQ > 100	21		21	10.75 (1.48)	10.32 (1.42)
Aggarwal and Gupta (2019)	Dynamic functional brain networks	fMRI	Multivariate graph learning	Resting-state brain networks	ASD: dynamic functional brain networks altered	ND: mean IQ > 100	Dataset 1	35	26	11.17 (1.49)	10.9 (1.62)
					ASD: alterations in multiple functional brain networks including cognitive control, subcortical, auditory, visual, bilateral limbic, and default-mode network.		Dataset 2	39	85	10.49 (1.53)	10.44 (1.49)
Green et al. (2019)	Neural Habituation and Generalization	fMRI	Sensory over-responsivity and brain response in sensory-limbic regions	Three fundamental stages of sensory processing: arousal (i.e., initial response), habituation (i.e., change in response over time), and generalization of response to novel stimuli	High_SOR_ASD: Reduced ability to maintain habituation in the amygdala and relevant sensory cortices and to maintain inhibition of irrelevant sensory cortices	ND full IQ	High_SOR	21	27	13.28 (3.35)	13.53 (2.79)
					Low-SOR_ASD: distinct, nontypical neural response patterns, including reduced responsiveness to novel but similar stimuli and increases in prefrontal-amygdala regulation across the sensory exposure	Verbal IQ High_SOR < TD*	Low_SOR	21		14.22 (2.32)	
Tietze et al. (2019)	Speech perception	fMRI	Audiovisual integration deficits in Aspergr syndrome	Semantic categorization task: disyllabic AV congruent and AV incongruent nouns	TD: stronger activation left auditory cortex (BA41)	ND: verbal IQ	16		16	39.50 (11.17)	33.75 (8.22)
Watanabe et al. (2019)	Neural timescale	fMRI	Intrinsic neural timescale	Resting-state networks (RSNs)	ASD + TD: similar whole-brain pattern of intrinsic neural timescales	ND	25		26	≥18 years old	
					Longer timescales in frontal and parietal cortices	Full/verbal/performance IQ ≥ 80					
					Shorter timescales in sensorimotor, visual, and auditory areas						
Lloyd-Fox et al. (2018)	Development	fNIRS	Prospective longitudinal study 36 months	Human vocalizations compared to non-vocal sounds	ASD: reduced activation to visual social stimuli across IFG and pSTS-TPJ	Developmental ability TD > ASD***	High-risk ASD	20	16	149.35 days (27.28)	153.81 (25.67)
			First months of life -> later developed ASD 3 years old		Reduced activation to vocal sounds and enhanced activation to non-vocal sounds within MTG-STG		ASD	5	16		
Millin et al. (2018)	Adaptation	fMRI	Auditory cortical adaptation	Repeated audiovisual stimulation in early sensory cortical areas	Initial transient responses equivalent ASD and TD	ND full-scale IQ > 100	24		29	23	23
					ASD: in auditory but not visual cortex, greater post-transient sustained response in the fixed-interval timing condition						
					ASD: individual differences in the sustained response in auditory cortex correlated with symptom severity						
Green et al. (2018)	SOR	fMRI	Aversive sensory stimuli and attentional modulation	Interpreting communicative intent: with and without a tactile sensory distracter, and with and without instructions directing their attention to relevant social cues	ASD: decreased activation in auditory language and frontal regions for task in the presence of the sensory distracter,	ND mean full-scale IQ > 100	15		16	14.09 (2.70)	14.97 (2.44)
					ASD: increased medial prefrontal activity during tactile stimulation						
Green et al. (2017)	Thalamocortical connectivity and SOR	fMRI	Role of pulvinar connectivity during mildly aversive sensory input		ASD: aberrant modulation of connectivity between pulvinar and cortex (including sensory-motor and prefrontal regions) during sensory stimulation	ND Mean Full-scale IQ > 100	19		19	13.71 (1.60)	13.61 (2.57)
					ASD: pulvinar-amygdala connectivity was correlated with severity of SOR symptoms						
Linke et al. (2018)	Connectivity	fMRI	Interhemispheric and thalamocortical functional connectivity	No task	Atypical processing of sounds related to social, cognitive, and communicative impairments	ND: mean IQ > 100	40		38	14.02 (2.76)	13.66 (2.65)
					ASD: severity of sensory processing deficits and lower verbal IQ related to reduced inter-hemispheric connectivity of auditory cortices						
					ASD: Increased connectivity between the thalamus and auditory cortex - associated with reduced cognitive and behavioral symptomatology						
Floris et al. (2016)	Structural lateralization	fMRI	Left and right-hemisphere specialization	Structural asymmetries in cortical regions of interest	ASD: stronger rightward lateralization within the inferior parietal lobule	ND mean full-scale IQ > 100	67		69	26.19 (6.79)	27.88 (5.99)
				Measures of language, motor, and visuospatial skills	ASD: reduced leftward lateralization extending along the auditory cortex comprising the planum temporale, Heschl’s gyrus, posterior supramarginal gyrus, and parietal operculum	Performance IQ TD > ASD**					
					More pronounced in ASD individuals with delayed language						
Green et al. (2016)	Salience Network Connectivity and SOR	fMRI	SOR symptoms related to salience network connectivity	Brain response to mildly aversive tactile and auditory stimuli	ASD: SOR related with increased resting-state functional connectivity between salience network nodes and brain regions implicated in primary sensory processing and attention	ND mean full-scale IQ > 100	28		33	12.95 (1.98)	12.93 (2.98)
				Resting-state salience network connectivity	ASD: strength of this connectivity at rest is related to extent of brain activity in response to auditory and tactile stimuli.						
Hoffmann et al. (2016)	Social perception network	fMRI	Activation and connectivity analyses	Face-, voice-, and audiovisual-processing brain regions	ASD: reduced connectivity between the left temporal voice area (TVA) and the superior and medial frontal gyrus	ND	10		20	32.22 (9.96)	31.11 (11.12)
					ASD: connectivity between the left TVA and the limbic lobe, anterior cingulate and the medial frontal gyrus as well as between the right TVA and the frontal lobe, anterior cingulate, limbic lobe and the caudate decreased with increasing symptom severity						
Schelinski et al. (2016)	Voice processing	fMRI	Voice processing	Vocal sound and voice-identity recognition	ASD: dysfunction in voice-sensitive regions during voice identity but not speech recognition in the right posterior superior temporal sulcus/ gyrus (STS/STG)	ND mean full-scale IQ > 100	16			33.75 (10.12)	33.69 (9.58)
					TD: right anterior STS/STG correlated with voice-identity recognition performance						
					ASD + TD: Passive listening to vocal, compared to non-vocal, sounds elicited typical responses in voice-sensitive regions						
Watanabe and Rees (2016)	Gray matter	MRI	Relative gray matter volumes (rGMVs)	Measure cortical networks, how they changed with age, and their relationship with core symptomatology.	ASD: age-associated atypical increases in rGMVs of auditory and visual networks	ND mean full-scale IQ > 100	Children	89	96	12.4 (3.0)	13.1 (2.6)
				Public neuroimaging data	ASD: age-related aberrant decrease in rGMV of a task-control system (fronto-parietal network, FPN)	ND mean full-scale IQ > 100	Adults	34	50	23.9 (5.5)	24.0 (5.0)
					Enlarged rGMV of the auditory network in ASD adults - associated with the severity of autistic socio-communicational core symptom						
					Visual network—correlated with the severity of restricted and repetitive behaviors						
Yamada et al. (2016)	Insular cortex	fMRI	Resting state	Sub-regional organization of the insula and the functional characteristics of each sub-region	ASD: alterations in the anterior sector of the left insula and the middle ventral sub-region of the right insula	ND mean full-scale IQ > 100	36	38		29.9 (7.1)	32.5 (7.3)
				Data-driven clustering analysis	TD: anterior sector of the left insula contained two functionally differentiated sub-regions for cognitive, sensorimotor, and emotional/affective functions						
					ASD: single functional cluster for cognitive and sensorimotor functions-anterior sector						
					ASD: volumetric increase right insula						

*AV* audiovisual integration, *MEG* magnetoencephalography, *MRI* magnetic resonance imaging, *MRS* Magnetic Resonance Spectroscopy, *TVA * “Temporal Voice Area”, *GAI* General Ability Index, *IFG* inferior frontal, *pSTS-TPJ* posterior temporal, *MTG-STG* left lateralised temporal, *BOLD* Blood Oxygenation Level Dependent, *SOR* Sensory over-responsivity, *Glu* Glutamate, *Glx* glutamine, *ND* no differences

**Table 12:** Summary of publications sources using several multimodal tools, organized by year

Source	Field	Method	Experiment				*n*			Mean age (SD)	
			Paradigm	Task/stimuli	Results	Cognitive standard scores Relevant results	ASD		TD	Or Mean age (min range- max range)	
										ASD	TD
Pierce et al. (2021)	Spontaneous brain activity	EEG-MRS	Resting-state alpha power	MRS protocol: [] excitatory (Glu + Glx) and inhibitory (GABA)	Decreased resting alpha power	> 100	31		31	11.3 (1.6)	10.6 (1.9)
	Neurochemical		Concentrations of excitatory and inhibitory neurotransmitters		ND: Glu						
					Glx in the temporal-parietal junction						
Roberts et al. (2020)	Structural and neurochemical factors	MEG	Identify and contrast the multiple physiological mechanisms	Sinusoidal tones of 500 Hz frequency (300 ms duration; 10 ms ramps) with a pseudo-randomized 600–2000-ms inter-trial interval were presented at 45-dB sensation level, after individual hearing threshold determination	Auditory radiation fractional anisotropy: predict 52% of M50 latency TD	Above the second percentile (SS > 70) on the non-verbal reasoning composite score of the cognitive assessment	77		40	11.4 (2.4)	11.5 (2.8)
	Brain’s response time to auditory tones	MRI	Associated with auditory processing efficiency		Auditory radiation fractional anisotropy: predict 12% of M50 latency ASD						
		GABA MRS			ASD: altered patterns of M50 latency modulation characterized by both higher variance and deviation from the expected structure–function relationship established with the TD group						
					TD M50 latency model identified subpopulation of ASD—outliers of TD						
					Subpopulation of ASD: unexpectedly long M50 latencies in conjunction with significantly lower GABA levels						
Bloy et al. (2019)	Lexical access	MEG	Neurophysiological marker of language ability	Words and plausible, pronounceable non-words	Integral of event-related desynchronization in the 5–20 Hz band during 0.2–1 s post auditory stimulation with interleaved word/non-word tokens	ND: IQ (mean) > 100	35		15	9.4 (1.1)	8.8 (1.4)
		Structural MRI			Correlation with clinical assessment of language function in both ASD and TD	Language ability: TD > ASD***					
					Not related to general cognitive ability nor autism symptom severity						
De Stefano et al. (2019)	Oscillatory activity in response to auditory stimuli	EEG	Drive the cortex to oscillate at a range of frequencies	Tone amplitude-modulated by a sinusoid linearly increasing in frequency from 0–100 Hz over 2 s	Older ASD: decreased ability to phase-lock to the stimulus in the low gamma frequency range	IQ > 90	Child	7	7	8.86 (1.77)	8.71 (1.50)
					ND between young ASD + TD		Adult	8	8	16.5 (4.14)	18.00 (4.90)
					Developmental trajectories: different for low gamma-power						
					TD show decrease gamma-power, while ASD did not						
					Low gamma STP: correlated with increased clinical scores for repetitive behaviors						
Borowiak et al. (2018)	Visual-speech recognition	fMRI	Extracting speech information from face movements	Lip reading; PPI analysis	ASD: decreased BOLD response during visual-speech recognition in the right visual area 5 (V5/MT) and left temporal visual-speech area (TVSA)	ND full-scale IQ > 85	17		17		
		Eye tracking			ASD: right V5/MT—positive correlation with visual-speech task						
					ASD: lower functional connectivity between the left TVSA and the bilateral V5/MT and between the right V5/MT and the left IFG						
					ASD and TD = similar responses in other speech-motor regions and their connectivity						
Tanigawa et al. (2018)	Language Processing	MRI	Surface-based morphometric structure analysis	Auditory word comprehension task	No structural differences	ND: mean IQ > 100	16		17	13.4 (1.1)	13.4 (1.2)
		MEG	Cortical responses		ASD: correlation between volume of the left ventral central sulcus (vCS) and linguistic scores						
					ASD: weaker cortical activation in the left vCS and superior temporal sulcus						
					ASD: atypical gamma-band (25–40 Hz) network centered on the left vCS						
Berman et al. (2016)	Integratation of diffusion MR measures of white-matter microstructure and MEG measures of cortical dynamics	MEG	Associations between brain structure and function within auditory and language systems	Diffusion MR tractography: delineate and quantitatively assess the auditory radiation and arcuate fasciculus segments of the auditory and language systems	ASD: Atypical development of white matter and cortical function	No reference of IQ	95		44	10.2 (2.6)	10.4 (2.4)
		Diffusion MRI			ASD: Atypical lateralization	Language impairment:					
					M100: marker of ASD severity; MMF delay: language impairment	LI: SS < 85					
						TD: SS > 70					
Port et al. (2016)	E/I balance and Gamma	MEG	MEG, MRI and MRS data	200, 300, 500, and 1000 Hz (300 ms duration; 10 ms ramps) sinusoidal tones	Auditory cortex localized phase-locked Gamma was compared to resting Superior Temporal Gyrus relative cortical GABA concentrations for both children/adolescents and adults	SS > 70	Children	27	11	11.7 (0.36)	10.6 (0.56)
		MRI			Children/adolescents_ASD: decreased GABA1/Creatine (Cr) levels, though typical Gamma		Adults	15	21	21.9 (1.1)	27.0 (1.2)
		MRS			Children/adolescents_ASD: lack of typical maturation of GABA1/Cr concentrations and gamma-band coherence						
					Children/adolescents_ASD: failed to exhibit the typical GABA1/Cr to gamma-band coherence association						
Sadeghi Bajestani et al. (2016)	Hemispheric asymmetry	EEG	Extracted two Indexes: Divergence (D) and number of Poincaré section points further from threshold	Animation with audio (V-A) for 5 min and watching the animation with muted audio band (VwA)	Hemispheric asymmetry in ASD children does not follow norm patterns	–	60		60	Range: 3–11	Range: 3–11
Edgar et al. (2015)	Maturation of auditory cortical responses	MEG	Auditory time-domain and time–frequency activity	Tones: 500- and 1000-Hz tones of 300-ms duration (binaurally)	ASD: right STG M100 latency delay	ND: IQ (mean) > 100	52		63	10.1 (1.7)	9.8 (1.8)
		MRI		T1-weighted structural MRI	Left and right STG: greater pre- to post-stimulus increase in 4- to 16-Hz TP for both tones in ASD versus TDC after 150 ms						
				Left and right 50-ms (M50), 100-ms (M100), and 200-ms (M200) time-domain and time–frequency measures (total power (TP) and inter-trial coherence (ITC))	Right STG: greater post-stimulus 4- to 16-Hz ITC for both tones was observed in TDC versus ASD after 200 ms						
					Age effects: left and right M200 decreasing with age in TDC but significantly less so in ASD						

*AV* audiovisual integration, *MEG* magnetoencephalography, *MRI* magnetic resonance imaging, *MRS* Magnetic Resonance Spectroscopy, *TVA * “Temporal Voice Area”, *GAI* General Ability Index, *IFG* inferior frontal, *pSTS-TPJ* posterior temporal, *MTG-STG* left lateralised temporal, *BOLD* Blood Oxygenation Level Dependent, *SOR* Sensory over-responsivity, *Glu* Glutamate, *Glx* glutamine, *ND* no differences

Magnetic resonance imaging (MRI) is an imaging technique that is used to assess the anatomy and physiology of brain circuits. Magnetoencephalography (MEG) is a functional neuroimaging technique that detects, records, and analyzes the magnetic fields produced by electrical currents occurring naturally in the brain (Cohen 1972). While EEG records brain electrical fields, MEG records magnetic fields. Similar to EEG and ERPs, MEG signals can be also time-locked to particular events, being called event-related magnetic fields (ERFs). As previously described for N100 (EEG signal), M100 (MEG) refers to a peak signal occurring at a latency of about 100 ms after stimulus onset. Both MEG and EEG are non-invasive methods for recording neural activity providing data with high temporal resolution (measured in milliseconds), thus providing unique information in terms of timing, synchrony, and connectivity of neural activity (Port et al. 2015). Despite the fact that EEG signals might display superimposed sources of activity, EEG is useful to quickly determine how brain activity can change in response to stimuli and to directly detect abnormal activity, having the advantage of being fully or semi-portable with an accessible cost for researchers. MEG has the advantage that the local variations in conductivity of different brain matter do not attenuate the signal, providing more accurate spatial resolution of neural activity than EEG (Landini et al. 2018). Regarding EEG and MEG use in young children, these two techniques offer advantages over some neuroimaging techniques, including fewer physical constraints and the absence of radiation and noise (Port et al. 2015). Still, EEG and MEG have limited spatial resolution, making it difficult to determine the precise location of neuronal activity with confidence. In contrast, MRI provides data with good spatial resolution, but lacks a good temporal resolution at the electrophysiological level and cannot provide frequency band discrimination. Functional magnetic resonance imaging (fMRI) uses MRI to measure the oxygenation of blood flowing near active neurons, being a valuable tool for delineating the human neural functional architecture (Cole et al. 2010). Combining EEG/MEG with MRI can increase the spatial resolution of electromagnetic source imaging, while tracing the rapid neural processes and information pathways within the brain (Liu et al. 2006), making them good candidates for multimodal integration.

#### Auditory evoked magnetic fields’ studies in ASD

Results from studies with high-functioning ASD individuals show delayed latencies at M50 and M100 auditory evoked responses, suggesting impairments at early auditory processes in the primary and secondary auditory cortex (Claesdotter-Knutsson et al. 2019; Matsuzaki et al. 2020; Roberts et al. 2019; Port et al. 2016; Edgar et al. 2013). It is thought that the major activity underlying M100 is located in the supratemporal plane, with superior temporal gyrus (STG) as the primer M50 generator (Edgar et al. 2015). STG results from the ASD group suggest increased pre-stimulus abnormalities across multiple frequencies with an inability to rapidly return to a resting state before the following stimulus (Edgar et al. 2013). Neurotypical individuals present a negative association between age and latency of M50 and M100 (Matsuzaki et al. 2020; Roberts et al. 2020), which indicates a functional decrease with age. Results show a similar pattern for children with ASD regarding M50, but not with M100 latencies (Matsuzaki et al. 2020). A group of ASD children and TD peers were compared at two time-points, from approximately 8 to 11 years old, showing M100 latency and gamma-band maturation rates similar between both groups (Port et al. 2016). A study of cascading effects on speech sound processing found lower brain synchronization in the early stage of the M100 component for ASD children (Brennan et al. 2016). A group of younger ASD children with approximately 5 years old was assessed in a different study, showing shorter M100 latencies in the left hemisphere (Yoshimura et al. 2021). The M200, considered an endogenous response associated with attention and cognition, seems to have a maximum amplitude around 8 years old, decaying with age. This pattern of age-dependent decrease in neurotypical children was less clear in the ASD group (Edgar et al. 2015), indicating perhaps a maturational delay.

Looking at data across lifespan from individuals with ASD without intellectual disability, delayed latencies were found above 10 years old *versus* shorter latencies in younger children, suggesting atypical brain maturation in ASD. Minimally verbal or non-verbal children with ASD (ASD-MVNV) seem to present greater latencies delays in M50 and M100 (components associated with language and communication skills) compared to ASD children without intellectual disabilities (Roberts et al. 2019). Similar association for verbal comprehension was found in the left auditory cortex regarding M200 latency response (Matsuzaki et al. 2017; Demopoulos et al. 2017). In auditory vowel-contrast mismatch field experiments (MMF), an association was found between MMF delay and language impairments in children with ASD (Berman et al. 2016). Furthermore, in a study testing pre-attentive discrimination of changes in speech tone, the amplitude of the early MMF component (100–200 ms) seems to be decreased in left temporal auditory areas for ASD children (Yoshimura et al. 2017). This group of ASD children, also diagnosed with speech delay, seem to have increased activity in the left frontal cortex compared to other ASD children without speech delay (Yoshimura et al. 2017). Deficits in auditory discrimination have also been reported in children with ASD, namely bilaterally delayed MMF latencies (Matsuzaki et al. 2019a) as well as rightward lateralization of MMF amplitude (Matsuzaki et al. 2019b) contrasting with the leftward lateralization found in NT children (Matsuzaki et al. 2019a). ASD children with abnormal auditory sensitivity seem to have longer temporal and frontal residual M100/MMF latencies (Matsuzaki et al. 2017). These findings were correlated with the severity of auditory sensitivity in temporal and frontal areas for both hemispheres (Matsuzaki et al. 2017). Taking these data together, ASD seems to be characterized by atypical neural activity in the auditory cortex, together with impaired auditory discrimination in brain areas related to attention and inhibitory processing. Such findings seem to be highly associated with language and comprehension deficits.

#### EEG and MEG frequency bands in auditory tasks

EEG and MEG can be transformed to decompose its raw signal into frequency band components. In adults, the typical frequency bands and their approximate spectral boundaries are delta (*δ*, from 1 to 3 Hz), theta (*θ*, from 4 to 7 Hz), alpha (*α*, from 8 to 12 Hz), beta (*β*, from 13 to 30 Hz), and gamma (*γ*, from 30 to 100 Hz) (Saby and Marshall 2012). Regarding ASD studies, decreased resting alpha power has been observed in children with ASD (Pierce et al. 2021). In noise experiment conditions, ASD children seem to have increased recruitment of neural resources, with reduced beta band top–down modulation (required to mitigate the impact of noise on auditory processing) (Mamashli et al. 2017). Interestingly, in quiet conditions, no differences were found between ASD and TD peers (Mamashli et al. 2017). A different study using sensory distracters (distracters that disrupts the processing of social cue interpretation) found decreased activation in auditory language and frontal regions in high-functioning youth with ASD (Green et al. 2018).

In auditory habituation studies measuring galvanic skin response, an aversion effect has been reported in ASD, likely due to sensory information overload. Results show consistent patterns of reduced habituation in ASD individuals, without the predicted steady decline that would be expected for habituation experiments. Instead, ASD adults showed a steady increase in the galvanic skin response over the course of the sessions (Gandhi et al. 2021). Regarding phase-lock auditory stimuli, no differences were found in the low gamma frequency range between ASD and TD children around 8 years old. A decrease in low gamma-power was observed in TD subjects around 17 years old but not in the ASD group (Stefano et al. 2019). The atypical gamma-band network in ASD seems to be located around the left ventral central sulcus (vCS) in children around 10 years old (Floris et al. 2016). Older ASD participants showed more pronounced low gamma deficits (Stefano et al. 2019), suggesting an increased background gamma-power that, similar to noise, can affect proper processing of stimuli. Interestingly, a recent study tested ASD children with and without atypical audiovisual behavior and found that only children with atypical audiovisual behavior showed increased theta to low gamma oscillatory power in the bilateral superior temporal sulcus and temporal region (Matsuzaki et al. 2022).

#### Cortical excitatory–inhibitory balance in ASD

Proper development of the central nervous system requires a fine balance between excitatory and inhibitory (E/I) neurotransmission. This E/I balance seems to be very important for cortical gamma-band activity, given that gamma waves are generated through connections between GABAergic inhibitory interneurons and excitatory pyramidal cells (Stefano et al. 2019; Port et al. 2017). A multimodal imaging study combined MEG, MRI, and GABA magnetic resonance spectroscopy (MRS) to assess physiological mechanisms associated with auditory processing efficiency in high-functioning children/adolescents with ASD. The study found longer M50 latency combined with decreased GABA in the left hemisphere for ASD individuals, suggesting an association between sensory response latency and synaptic activity (Roberts et al. 2020). Similar results were shown in a multimodal study that assessed cortical GABA concentrations and gamma-band coherence in the auditory cortex and superior temporal gyrus. Decreased GABA1/Creatine levels were found in children/adolescents with ASD, without the gamma-band coherence association typically seen in neurotypical subjects (Port et al. 2017). A different study combining EEG and MRS showed reduced glutamine in the temporal–parietal cortex associated with greater hypersensibility to sensory input detection (Pierce et al. 2021). A post-mortem study assessed the cytoarchitecture of the anterior superior temporal area (area of Brodmann), involved in auditory processing and social cognition, by quantifying the number and soma volume of pyramidal neurons in the supragranular and infragranular layers (Kim et al. 2015). Results showed no differences between ASD adolescents and adults age-matched with a neurotypical group (Kim et al. 2015). A different study looked at cortical neural inhibition for auditory, visual, and motor stimulation (Murray et al. 2020). Results showed no increase in the initial transient response in ASD individuals, with widespread changes in stimulus offset responses (Murray et al. 2020). Although results show similar patterns of transient response in ASD and TD groups for all cortical regions, larger fMRI amplitudes were found at later response components, approximately 6–8 s after stimulus presentation (stimulus duration of 20 s) (Murray et al. 2020). These studies suggest cortical excitatory–inhibitory imbalance in areas related to auditory processing in subjects with ASD.

#### Auditory sensory sensitivities in ASD

Sensory sensitivities can be assessed at three different stages of sensory processing: initial response to the stimuli, habituation, and generalization of response to novel stimuli. Brain imaging studies in individuals with ASD have found auditory discrimination deficits (Matsuzaki et al. 2019a; Abrams et al. 2019), as well as increased neural responsiveness upon repeated stimuli, with larger fMRI response in the auditory cortex that seems specific to temporal patterns of stimulation (Millin et al. 2018). Children with high sensory over-responsivity showed reduced ability to maintain habituation in the amygdala (Green et al. 2019), together with increased resting-state functional connectivity between salience network nodes and brain regions implicated in primary sensory processing and attention (Green et al. 2018). Children with low sensory over-responsivity showed atypical neural response patterns, with increased prefrontal–amygdala regulation across sensory exposure (Green et al. 2019). A different study found intact temporal prediction responses with altered neural entrainment and anticipatory processes in children with ASD (Beker et al. 2021). These results might explain atypical behavioral responses observed in ASD during sensory processing, mediated by top–down regulatory mechanisms.

Differences in response to sound familiarity have also been reported in children with ASD, with reduced activity in right-hemisphere planum polare for unfamiliar voices, reduced activity in a broad extent of fusiform gyrus bilaterally, and less activity in the right-hemisphere posterior hippocampus (Abrams et al. 2019). In a different fMRI study, the authors compared brain responses to vocal changes with different levels of stimulus saliency (deviancy or novelty) and different emotional content (neutral, angry) (Charpentier et al. 2020). Results show no differences between ASD and neurotypical adults regarding vocal stimuli and novelty processing, independently of emotional content. Brain processing appears typical in both groups, with activation in the superior temporal gyrus, and with larger activation for emotional compared to neutral prosody in the right hemisphere (Charpentier et al. 2020). These results suggest that the processing of emotional cues may be placed at later processing stages, such as insular activation, or at the hippocampus level. Interestingly, a recent study show altered voice processing in ASD which seems to be present already at the midbrain level of the auditory pathway (Schelinski et al. 2022).

Deficits in auditory discrimination have also been found both in MEG and brain imaging studies (Ganesan et al. 2016; Claesdotter-Knutsson et al. 2019; Abrams et al. 2019). Individuals diagnosed with ASD seem to have deficits in predicting the offset of standard tones, engaging less resources for duration tasks compared with pitch discrimination tasks (Lambrechts et al. 2018). Spectrally complex sounds can trigger two continuous neural MEG responses in the auditory cortex: the auditory steady-state response (ASSR) at the frequency of stimulation, and the sustained deflection of the magnetic field (sustained field).

The ASSR is an oscillatory response phased-locked to the onset of the stimulus, where the frequency of stimulation is represented by the same frequency in the primary auditory cortex (Stroganova et al. 2020). Both ASD and neurotypical children with 5–6 years old seem to show right dominant 40 Hz ASSR (Ono et al. 2020). No differences were found regarding neural synchrony at 20 Hz for both ASD and TD children between 5 and 12 years old (Stroganova et al. 2020; Ono et al. 2020), suggesting a normal maturation of ASSR for low frequencies. Interestingly, the right-side 40 Hz ASSR increased with age in the neurotypical group, as opposed to ASD children (Ono et al. 2020). Reduced 40 Hz power was also found in adolescents with ASD at both right and left primary auditory cortex, with no difference in gamma-band responses (Seymour et al. 2021). Diminished auditory gamma-band responses were found in ASD children, indicating that peak frequencies likely vary with developmental age (Roberts et al. 2021).

The sustained field (SF) is a baseline shift in the electrical and magnetic signals upon exposure to a sound lasting for several seconds. The SF adapts to the probability of a sound pattern, reflecting the integration of pitch information across frequencies within the tonotopic map of the primary auditory cortex (A1) (Stroganova et al. 2020). Children diagnosed with ASD seem to have atypical higher order processing in the left hemisphere of the auditory cortex, with cortical sources of SF located in the left and right Heschl’s gyri (primary auditory cortex) (Stroganova et al. 2020).

The severity of sensory processing deficits in ASD also seems to be correlated with reduced inter-hemispheric connectivity of auditory cortices (Linke et al. 2018; Tanigawa et al. 2018) and lower verbal IQ (Linke et al. 2018). Interestingly, increased connectivity between the thalamus and the auditory cortex was found in patients with reduced cognitive and behavioral symptomatology (Linke et al. 2018; Tanigawa et al. 2018), which suggests high thalamocortical connectivity as a potential compensatory mechanism in ASD (Linke et al. 2018). Individuals diagnosed with ASD also seem to present abnormal modulation of connectivity between pulvinar and cortex, with greater increases in pulvinar connectivity with the amygdala (Green et al. 2017). Regarding processing of social stimuli, reduced connectivity between the left temporal voice area and the superior and medial frontal gyrus was found in ASD patients (Hoffmann et al. 2016). Decreased connectivity between the left TVA and the limbic lobe, anterior cingulate and the medial frontal gyrus as well as between the right TVA and the frontal lobe, anterior cingulate, limbic lobe and the caudate seems to be associated with increased symptom severity (Hoffmann et al. 2016).

#### Language and speech processing in ASD

Language acquisition involves the integration of top–down and bottom–up processes. Language deficits present in ASD diagnostic criteria may be related to one of these integration processes or a combination of both. ASD individuals can have sensory deficits in the bottom–up early sensory processing and/or prediction deficits related to higher order assessment.

Phonological processing seems to be disrupted in ASD children, displaying attenuated MEG response in the right auditory cortex to both legal and illegal phonotactic sequences (Brennan et al. 2016). Interestingly, ASD children do not seem to have differences in phonological competence but significantly differ in other measures, such as attention, syntax, and pragmatics (Brennan et al. 2016; Wagley et al. 2020), suggesting impairments at language structure knowledge. An event-related desynchrony of the auditory cortex in the 5–20 Hz range seems to index language ability in both children with ASD and neurotypical controls (Bloy et al. 2019). When speech and non-speech were compared, ASD children with poorer language composite scores presented a general auditory processing deficit, with atypical left hemisphere responses in the high order time-window of 200–400 ms (Yau et al. 2016), and different neural and behavioral effects of syllable-to-syllable processing in speech segmentation (Wagley et al. 2020).

When considering visual-speech recognition tasks, ASD individuals seem to have difficulties in extracting speech information from face movements (Borowiak et al. 2018). Decreased Blood Oxygenation Level Dependent (BOLD) responses were detected in the right visual area 5 and left temporal visual-speech area, as well as lower functional connectivity between these two brain regions implicated in visual-speech perception (Borowiak et al. 2018). This multimodal fMRI study combined with eye-tracking data showed that the ASD group had reduced responses not only for emotional but also neutral facial movements (Borowiak et al. 2018). High-functioning adults with ASD seem to have typical responses for voice-identity tasks, and dysfunctional speech recognition in the right posterior temporal sulcus (Schelinski et al. 2016).

Predictive processing was tested in a naturistic environment, by measuring surprisal values, or how much information of word contributes given some linguist context (Brennan et al. 2019). Results showed bilateral temporal effect for sentence-context linguistic predictions in an early time-window (from 26 to 254 ms), for both high-functioning ASD children and TD peers with 3–6 years old (Brennan et al. 2019). A different study showed that as surprisal values increase, the amplitude of the neural response also increases in the left primary auditory cortex, posterior superior temporal gyrus (pSTG), and inferior frontal gyrus (IFG) in neurotypical children (Wagley et al. 2020). However, ASD children with lower IQ levels did not display such learning pattern (Wagley et al. 2020).

A longitudinal study tested children in the first months of life (around 4 months old) who later developed ASD, at 3 years old (Lloyd-Fox et al. 2018). Using functional near-infrared spectroscopy (fNIRS), infants later diagnosed with ASD showed reduced activation to visual social stimuli across the inferior frontal (IFG) and posterior temporal (pSTS-TPJ) regions of the cortex, reduced activation to vocal sounds, and enhanced activation to non-vocal sounds within left lateralized temporal regions (Lloyd-Fox et al. 2018). These results suggest that atypical ASD cortical responses may be detectable at early stages.

#### A right ASD brain? Lateralization and inter-hemispheric connectivity

The inferior frontal and superior temporal areas in the left hemisphere are crucial for human language processing (Yoshimura et al. 2017). Some striking findings report ASD group differences in left and right hemispheres (Edgar et al. 2013; Sadeghi Bajestani et al. 2017). Results show lower right–left hemispheric synchronization in young children with ASD (Brennan et al. 2016), stronger rightward lateralization within the inferior parietal lobule, and reduced leftward lateralization extending the auditory cortex (Floris et al. 2016). Neurotypical peers show stronger activation in the left auditory cortex for semantic categorization tasks (Tietze et al. 2019), and hemispheric advantage that seems to be absent in ASD children (Edgar et al. 2015). Adolescents diagnosed with ASD show weaker cortical activation in the left ventral central sulcus at word comprehension tasks (Tanigawa et al. 2018), indicating atypical hemispheric functional asymmetries.

#### Resting-sate studies of the auditory network in patients with ASD

Resting-state connectivity is a correlated signal between functionally related brain regions in the absence of any stimulus (spontaneous signal fluctuation). Results show dynamic functional brain networks altered in children diagnosed with ASD, including cognitive control, subcortical, auditory, visual, bilateral limbic, and default-mode network (Stickel et al. 2019). High-functional ASD adults also present alterations in the anterior sector of the left insula and the middle ventral sub-region of the right insula in the ASD brain (Yamada et al. 2016). Atypical spread of activity seems to be present in ASD individuals, indicated by altered dynamic lag patterns in salience, executive, visual, and default-mode networks (Raatikainen et al. 2020). Regarding intrinsic neural timescales, both high-functional adults with ASD and TD peers presented longer timescales in frontal and parietal cortices and shorter timescales in sensorimotor, visual, and auditory areas (Watanabe et al. 2019). Individuals with ASD also seem to display a volumetric increase in the right insula (Yamada et al. 2016). Given that the right insula is primarily specialized for sensory and auditory-related functions, such volumetric expansion might be functionally correlated with previously reported ASD alterations in auditory stimuli processing and auditory sensitivity.

#### Diffusion MRI studies of the auditory network in patients with ASD

A recent study in children with ASD reports decreased gray matter volume at the fronto-parietal network, associated with the severity of communication score and restricted behaviors. In adults, an increase of gray matter was reported at the regions of auditory and visual networks, with the auditory network being correlated with the severity of the communication core, and visual networks with severity of repetitive and restricted behavior (Yamada et al. 2016). Uncoupled structure–function relationships in both auditory and language networks have also been reported in ASD (Berman et al. 2016), with changes in the relative weight of white matter contribution to structure–function relationships (Roberts et al. 2020).

## Final remarks

### Experimental design in ASD studies

Despite a large number of studies assessing autism spectrum disorder at the level of auditory processing, it is still not possible to conclude the cause of auditory symptomatology present in ASD. Children and adults diagnosed with ASD have a neurodivergent cognitive profile, and the heterogeneity of both severity and type of symptoms, probably contributes for this lack of causal explanation. Another heterogeneity factor comes from the inclusion criteria for ASD participants, such as the diagnostic assessment confirmation tools or the measurements of IQ levels. Some studies analyze their data considering verbal and non-verbal abilities for ASD children, while other studies refer only to the full performance of IQ levels. To better illustrate this issue, especially for language components assessment, we can look at the study performed by Bloy et al. (2019) that found no differences in IQ mean values between ASD and TD peers, but highly significant differences in language ability. Future studies should include a wider range of standardized assessment tools to determine discrepancies in autistic traits, and larger sample size to avoid limitations in the assessment of within-group and multiple comparisons.

A consideration should also be made about the accuracy of diagnosis of children with ASD. In a longitudinal study, Port and colleagues referred to the inclusion of a group of children with approximately 8 years old considered to be initially on the spectrum. A later follow-up revealed that these children exhibited optimal outcomes at approximately 12 years old, no longer meeting diagnostic criteria for ASD (Port et al. 2016). Interestingly, these children showed results in-between ASD and TD peers. A similar in-between result was found in ASD siblings emotional perceptions (Waddington et al. 2018), indicating a possibility of contextual interference in ASD traits, such as parenting. Sometimes, parents’ perception questionnaires are assessed without taking into consideration the social, economic, and affective influence of parenting and/or children’s educators and peers. When designing auditory research studies, it would be important to also consider other relevant daily-life features, such as special talents, levels of anxiety and aggression, and sensory challenges.

One main limitation of brain imaging and EEG auditory studies in ASD is individual discomfort thresholds, such as sensibility to sound levels or immobility requirements during imaging sessions. By analyzing experimental designs, it is possible to see the wide variety of methods and protocols that are applied, even for similar techniques. For instance, some participants are allowed to sleep during ABR recordings (Fujihira et al. 2021), while some participants watch videos during the experiment (Jones et al. 2020; Chen et al. 2019), creating different conditions to assess similar variables across studies. Another important consideration is medication status as an eligibility criterion for ASD studies. Some studies detail the presence or absence of medication, such as risperidone, anxiolytics, or antidepressants (Port et al. 2016), or simply exclude ASD-medicated participants, while other do not report any criteria regarding medication.

The underlying conceptualization of autism and associated deficits and impairments through the lens of the DSM might push researchers and clinicians to prioritize the correction of perceived deficits as the major goal of behavioral intervention (American Psychiatric Association (APA) 2013; Schuck et al. 2022). Given the neurodiversity and individual differences present in ASD subjects, future studies should ensure a comprehensive neuropsychological evaluation, for both ASD and control subjects. As an example, studies often include the term “high or low functioning”, which is not an official medical diagnosis; hence, it is not clear at every study whether this descriptor is based on full-scale IQ and language, verbal and non-verbal, or adaptive behavior and daily functioning measures. To acknowledge cultural differences, future studies could also include social and ecological validity measures (Schuck et al. 2022), with assessment of the socio-cultural context at individual, institutional, and family levels, using self-report measures. For a better understanding of the human neurocognitive spectrum, both ASD and control subjects should be assessed at other areas of interest, such as self-determination, self-esteem, social inclusion, well-being, personal development, interpersonal relationships, measures of quality of life, and functional adaptive skills (Schuck et al. 2022).

### Auditory cortex: a central role in ASD?

#### Deficits in bottom–up early sensory processing of auditory input

Low-level auditory stimuli (e.g., pitch discrimination) deficits vary widely within ASD, with some interesting differences when low- and high-functioning children (based on IQ assessment) are compared (Table 13). ASD individuals seem better at detecting additional unexpected and expected sounds at several acoustic parameters, showing an increased auditory perceptual capacity, but display deficits in detection and discriminatory tasks, especially in the presence of noise. Difficulties in the processing of sensory stimuli have been confirmed by electrophysiology and brain imaging data at early stages of perceptual processing. ASD patients with verbal disabilities seem to be particularly impaired in terms of cortical processing of acoustic inputs. Larger idiosyncrasy seems to be present in high-level auditory areas, with deficits being associated with severity of social and communication symptoms. One possibility is to look at the core aspects of autism as a cascading effect of unusual auditory and language trajectories (prosody, semantic context). Individuals with ASD seem to have alterations in multisensory integration, displaying poorer performance in multisensory tasks requiring the auditory modality. It is still not clear whether ASD sensory idiosyncrasy affects all types of audiovisual integration and whether these deficits can be compensated by later attentional processes. For non-multimodal studies, further auditory investigation should guarantee that unisensory conditions can be assessed without visual influence (blind conditions) and should control whether auditory processing responses are affected by attention deficits.

**Table 13:** Integrity of cognitive function

Main results			
**Attention**	*Deficits in divided, sustained, selective, and spatial attention*		
	*ND when types of attention are compared*		
	**HIGH-FUNCTIONING**		
	Attenuated P3a mean voltages		
	ADULTS: ND P3a latencies		
	ADULTS: Early ERP responses (P50 amplitude) positively correlated to increased sensory sensitivity		
**MSI**	*Distinctive in ASD*		
	**LOW-FUNCTIONING**		**HIGH-FUNCTIONING**
	Impairments at sensitivity to asynchronies (video-audio)		**Low-level perceptual processes**
	Correlated with language abilities		Intact low-level audiovisual integration
			Greater multisensory reaction time facilitation for TD adults
			***High-level perceptual processes***
			Poorer multisensory temporal acuity
			Poorest performances in auditory modality compared to visual modality
**Acoustic parameters**	**HIGH-FUNCTIONING**		
	Deficits in the intensity or loudness of stimuli		
	Deficits in discrimination (e.g., higher auditory duration discrimination threshold of stimuli)		
	Enhanced memory for vocal melodies		
	Enhanced pitch discrimination		–
	Discrimination ability varies widely within ASD		
**Noise**	Detrimental effect of noise and increased arousal		
	Worse speech comprehension in noise condition		
	ND: without noise condition		
**Temporal processing silent gaps**	Reduced P2 amplitude		
**Sensory habituation**	**LOW-FUNCTIONING**		**HIGH-FUNCTIONING**
	Reduced habituation P1		No reduction between the first and the last ERP
	Decrease in the amplitude MMN		TD: negative slope; SD: positive slope
**Environmental change**	**LOW-FUNCTIONING**		
	Greater P3a amplitude to novel sounds		
	Youth: slower attenuation of the N1 response to infrequent tones and P3a response to novel sounds		
**Hemisphere activation**	**LOW-FUNCTIONING**		**HIGH-FUNCTIONING**
	Speech detection left hemisphere		Bilateral attenuation
			Negative MMN response right hemisphere more activated
**Error prediction**	**HIGH-FUNCTIONING**		
	ASD + TD: decreased MMN		
	ND between ASD + TD		
	N1: similar for ASD and TD		
	MMN modulated by global context: smaller effect in ASD		
	No differences P3b		
	Reduced superior frontal cortex (FC) to unexpected events		
	Increased dorsolateral prefrontal cortex (PFC) activation to expected events		
**LANGUAGE**			
**Prosody**	**LOW-FUNCTIONING**		**HIGH-FUNCTIONING**
	Reduced P1 amplitude		diminished amplitude of P3a
	Larger P3a amplitude (P3a latencies shorter in adults)		slower perceptual discrimination
	Earlier MMN		
**Vowel + tone**	Pure-tone: diminished response amplitudes and delayed latency MMN; ND P3a		
	Vowel: smaller P3a; ND MMN		
**Speech vs. non-speech**	**LOW-FUNCTIONING**		**HIGH-FUNCTIONING**
	Speech stimuli	No P1 enhancement as TD	N330 match/mismatch responses right hemisphere
	Nonspeech stimuli	Similar P1 enhancement	TD: larger MMN responses (speech pitch contour) and stronger MMN (speech pitch height)
	Nonvocal sounds	Smaller P100	
		Smaller right fronto-temporal negative Tb peak non-vocal sounds	ASD: more positive MMR (speech pitch height)
		Atypical response to non-vocal sounds	
**Semantic congruence**	**LOW-FUNCTIONING**		
	N400 effect with shorter latency in TD		
	Delayed speed of processing		
**SON**	**LOW-FUNCTIONING**		**HIGH-FUNCTIONING**
	N100 amplitude, SON negativity		Auditory filtering disruption
			Strength of LPPs positively correlated with auditory filtering abilities
			TDs and ASD-Vs: significant MMRs to OON multisetting

Note: descriptor of functioning (low versus high) based on IQ measurements

Difficulties in understanding others are a core feature of autism spectrum disorders (Baron-Cohen 2001). At the emotional level, individuals with ASD seem to have deficits judging auditory valence and vocal emotion recognition, but with normal pro-social functioning, suggesting a normal social awareness and motivation. Future auditory studies should also look into Theory of Mind manifestations, emphasizing social motivation and reward associated with communication. A detrimental effect on communication and increased arousal on ASD children is observed in experiments within a noise context. Interestingly, when the context changes to silent, no differences in speech comprehension are found, suggesting a relevant role of auditory sensory overload in ASD individuals.

#### Changes in higher-order integration processing of auditory information

Results assessing phonological processing, language components, speech non-speech, and visual-speech recognition, suggest that processing at the auditory cortex is altered in ASD. This atypical sensory processing may interfere with perceptual mechanisms where individuals anticipate what will happen, based on their perceived sensory information. Observed differences in auditory perception in ASD might not be related with attention allocation to acoustic stimuli, but rather to difficulties in recognition and integration of acoustic information, such as the process of understanding speech from other people. Deficits at the level of communicative function would reduce the ability of individuals with ASD to learn several language components, such as phonology, syntax, and semantics. Children use prior information to incrementally narrow down the set of possible interpretations for a sentence, highlight how high-level representations can propagate to low-level processing stages. Deficits in habituation or adaption could potentially lead to an inability to form predictions. Such changes in higher order processing may impact the development of language and interfere with communication.

Integrity of the central nervous system seems to be affected in ASD individuals, with auditory alterations being detectable during early development (Table 14). Several studies indicate atypical brain maturation, abnormal neural network synchronization, and functional alterations in the primary auditory cortex, trailed by impaired cognitive function, such as attention, inhibitory processing, and neural discrimination processes. Most individuals diagnosed with ASD have auditory sensory issues, being mainly hypersensitive to sounds. Some reports also indicate that individuals with ASD may have deficits in lateralized cognitive functions as well as functional and brain structural asymmetry, with disproportionate overgrowth of audio and visual sensory networks. In social tasks involving auditory processing of verbal cues, a reduced connectivity between the left temporal voice area and the superior and medial frontal gyrus has been reported. Interestingly, thalamocortical overconnectivity has been reported in several studies albeit with different interpretations: it might reflect lack of thalamocortical inhibition (which could cause difficulties in attentional sensory information), or it might be a compensatory mechanism (that could serve to mitigate reduced synchronization).

**Table 14:** Integrity of central nervous system

Field	Major results and conclusions		
**Evoked-magnetic fields**	**50 and 100**	*High-functioning ASD*	
		Impairments at early auditory processes	
		Delayed M50 and M100 latencies children and adults (> 10 years)	
		Short P100 young children (~ 5 years)	
	**200**	Atypical brain maturation	
		M200 lower decrease with age	
		Left temporal auditory areas	
	**MMF delay**	Language	
		Sound discrimination	ASD: Bilateral
		Independent of cognitive performance	TD: leftward lateralization of MMF amplitude
		Impaired neural discrimination	ASD: rightward lateralization MMF amplitude
		Inhibitory processing	
**Noise/distractor**	Abnormal auditory sensitivity		
	Quiet condition: common neural sources		
	Noise condition: MMF response in the right IFG was preserved in the TD group, but reduced relative to the quiet condition in ASD group		
	Noise: reduced normalized coherence in the beta band (14–25 Hz) between left temporal and left inferior frontal sub-regions		
**Frequency bands**	**α **decreased resting alpha power		
	**β **reduction in top–down modulations		
	**γ **ND young		
	Older ASD: deficits low gamma		
	→ E/I imbalance → critical period		
**E/I balance**	Reduced glutamine in the temporal-parietal cortex		
	Decrease GABA in the left hemisphere		
	Number and soma volume of pyramidal neurons		
	Inhibition disruption: ND early response		
	Increases in cortical neural excitability after stimulus offset		
**Attention**	Inability to extract the temporal regularities of the stimulation sequence		
	**Neural responsiveness**	Larger fMRI response in the auditory cortex	
		Reduced ability to maintain habituation in the amygdala	
		Increased resting-state functional connectivity between salience network nodes and brain regions implicated in primary sensory processing and attention	
**Voice**	Emotional vs. neutral change	Typical brain processing	
	Unfamiliar voices	Reduced activity in right-hemisphere planum polare	Area of auditory association cortex within the superior temporal gyrus
	Mother’s voice	Fusiform gyrus	Left-hemisphere occipital regions Temporal occipital regions in the right-hemisphere
**Pitch processing**	Atypical processing at the level of the core auditory cortex of the left hemisphere		
	**SF**	Left and right Heschl’s gyri, anterolateral to ASSR	
	**ASSR**	Low consistency of phase dynamics in A1 over time	ND 20 Hz
		Brain responses locally dysregulated	Reduced 40 Hz ASD (γ)
**Language**	**Phonological processing**	Impaired (attention, syntax and pragmatics)	
		Stronger rightward lateralization	Inferior parietal lobule
		Reduced leftward lateralization	Auditory cortex
	**Speech and non-speech**	General auditory processing deficit	*Planum temporale, Heschl’s gyrus, posterior supramarginal gyrus, and parietal operculum*
		Atypical left hemisphere responses (200-400 ms)	Weaker cortical activation in the left ventral central sulcus
	**Speech recognition**	Dysfunction in the right posterior temporal sulcus	Weaker activation in the left auditory cortex for semantic categorization tasks
	**Visual-speech recognition**	Difficulties in extracting speech information from face	↓ BOLD right visual area 5 (V5/MT)
		Emotional and neutral facial movement both impaired	↓ BOLD left temporal visual-speech area (TVSA)
		ND process of attention allocation (similar eye movement patterns)	
	**Predictive mechanisms**	High_functioning: ND	
		Low_functioning: different pattern of TD	
	**Lexical stress**	High-functioning ASD adolescents: right hemisphere is more activated than the left hemisphere	
**Resting state**	Children: altered dynamic functional brain networks		
	Adults: alterations in the functional organization of the left and right insular sub-regions		
	Volumetric increase in the right insula		
**Diffusion**	**Gray matter**	Increase volume with age at auditory cortex	
		Decrease volume task-control system	
	**White matter**	Uncoupled structure–function relationships in both auditory and language systems	
**Connectivity**	Interhemispheric connectivity increased between the thalamus and auditory cortex		
	Thalamocortical overconnectivity		
	Social processing: reduced connectivity between left temporal voice area and the superior and medial frontal gyrus		

*ND*  no differences between ASD and TD peer

#### Auditory processing in ASD and neurodevelopmental trajectories

Clinical biomarkers can offer the opportunity to improve predictions, diagnosis, stratification by severity and subtypes, and response indices for pharmaceutical development. Such biomarkers should ideally be robust, sensitive, specific to the disorder, and scale with severity (Port et al. 2015), but finding biomarkers for ASD requires a deep understanding of its neurobiological underpinnings. A correlation between neuroanatomical, genetic, biochemical, and immune findings with clinical ASD diagnosis is still unclear (Levin and Nelson 2015). Furthermore, the heterogeneity of age-related phenotypes in ASD poses a challenge in the pursuit of clinical biomarkers. In that sense, functional signatures derived from electrophysiological and imaging studies might become promising biomarkers given that they offer a temporal layer that could help revealing putative biological trajectories along the development process.

The brain is remarkably malleable, capable of restructuring itself in response to experience. Major sculpting of brain circuits occurs during specific time windows known as critical periods (Leblanc and Fagiolini 2011). Distinct critical periods underlie different modalities, ranging from visual processing to language and social development. Critical periods begin in primary sensory areas and occur sequentially in the brain, requiring a precise balance of excitatory/inhibitory (E/I) neurotransmission (Bourgeron 2009). These periods close after structural consolidation, diminishing future plasticity as the brain reaches adulthood. Although critical periods provide an exceptional time-window for learning and consolidation, they also represent a period of great vulnerability for the developing brain.

Different neurodevelopmental trajectories and brain maturation processes may explain the heterogeneity of behavioral and cognitive traits observed in ASD. Checking the integrity of the peripheral auditory system as a longitudinal measure of auditory maturation seems to hold some predictive value. ABR studies (both click-ABR and speech-ABR) tend to show longer latencies in children with ASD, and several EEG studies show unstable neural tracking in subcortical auditory processing. However, not all children have the same clinical presentation, suggesting the potential existence of auditory processing-related subtypes of children with ASD. Considering the results from ABR studies, one interesting finding is ABR longitudinal profile across development that seems to indicate shorter latencies in newborns, longer latencies in ASD children compared to newborns, no differences in adolescents, and normal ABR in adults. Infants and toddlers later diagnosed with ASD present delayed auditory brainstem responses, mainly with later wave V latencies (Tecoulesco et al. 2020; Ramezani et al. 2019; Chen et al. 2019), that does not seem to persist through development. These findings highlight a potential difference in terms of auditory brainstem maturation timing, or the existence of compensatory mechanisms such as increasing myelin density. Given that ABR testing is relatively non-invasive and low cost, it could be advantageous to study longitudinal auditory brainstem maturation in children at a higher risk for ASD.

Interestingly, diffusion MRI and MEG studies suggest a deficiency in audiovisual temporal processing, with prolonged cortical response in older children with ASD, mainly mismatch negativity and middle latency delays (M50/M100) (Stevenson et al. 2017, 2018), together with atypical development of white matter and cortical function (Berman et al. 2016). Such results could reflect an abnormal maturation of the brainstem that could affect the temporal synchrony of neuronal firing, or/and white matter alterations that could lead to poor signal conduction. Given the range of ASD presentations along development, it could be useful to assess the longitudinal profile of ASD children in a multimodal analysis, comparing the neuropsychological profile, the auditory brainstem maturation, together with an assessment of myelin and cortical response profiles.

The usefulness of longitudinal EEG as a diagnostic tool for uncovering developmental trajectories in ASD has been recently suggested, showing that delta and gamma frequency power trajectories can differentiate autism outcomes, in the first postnatal year (Gabard-Durnam et al. 2019). Furthermore, EEG spectral power has been suggested as a marker to differentiate between low- and high-risk ASD infants at 6 months of age (Tierney et al. 2012), as well as a marker of disease severity in girls with Rett syndrome (Roche et al. 2019). The observation of increased power in the delta band could represent abnormal cortical inhibition due to dysfunctional GABAergic signaling (Roche et al. 2019). Given that auditory-related gamma and alpha powers also seem to be altered in ASD subjects along development (Port et al. 2016; Pierce et al. 2021; Stefano et al. 2019), it would be interesting to assess these measures together with social, cognitive, and language abilities, as potential predictors of later outcomes in ASD.

Not many studies have looked into E/I balance related to auditory processing in ASD. The imbalance hypothesis is highly promising, given the suggestion of cortical excitatory–inhibitory imbalance in areas related to auditory processing in subjects with ASD (Murray et al. 2020). It is also highly promising, since it provides the basis for a number of ASD biomarkers at various levels, from molecules to the neural networks that ultimately determine behavior (Levin and Nelson 2015). For instance, positron emission tomography (PET) is a molecular imaging technique that can be utilized in vivo for dynamic and quantitative measurement of neurotransmitter release in the human brain. Given the clear evidence stemming from animal studies (Castro and Monteiro 2022) showing auditory dysfunction and E/I imbalance in different animal models of ASD, it would be interesting to see results from PET studies looking at excitatory and inhibitory neurotransmitter systems, cerebral glucose metabolism, blood flow perfusion, and inflammation in the CNS in ASD patients, while performing specific auditory tasks for assessment of sensory function. In summary, a multivariate combination of biomarkers may be the most promising tool for a better understanding of the significant role of neurodevelopmental change in ASD.

## Data Availability

All data is provided with this paper.
